# Supplementary material for: Reciprocal carbonyl–carbonyl interactions in small molecules and proteins
Source: Nat Commun. 2017 Jul 19;8:78. doi: 10.1038/s41467-017-00081-x (PMC5517579; doi:10.1038/s41467-017-00081-x)
Supplement: Supplementary file 1 — Supplementary Information [file 41467_2017_81_MOESM1_ESM.pdf]

SI GUIDE

File Name: Supplementary Information
File Description: Supplementary Figures, Supplementary Tables and Supplementary Methods.

Supplementary Data

File Name: Supplementary Data 1
File Description: Cartesian coordinates of compounds 1-8 optimized at B3LYP/6-311+G(2d,p) level of theory by freezing dihedral angles of the twisted conformation to the X-ray values as shown in Supplementary Figure 2. [Note that Compounds 1 and 4 gave negative frequencies after constrained geometry optimization. We eliminated the negative frequency by reoptimization during which there are small deviations in the frozen dihedral angles from their X-ray values].

File Name: Supplementary Data 2
File Description: 2184 PDB structures ranked by frequency of reciprocal C=O···C=O interactions in them.

File Name: Supplementary Data 3
Description: cif file of compound 2

File Name: Supplementary Data 4
Description: cif file of compound 3

File Name: Supplementary Data 5
Description: cif file of compound 4

File Name: Supplementary Data 6
Description: cif file of compound 5

File Name: Supplementary Data 7
Description: cif file of compound 6

File Name: Supplementary Data 8
Description: cif file of compound 7

File Name: Supplementary Data 9
Description: cif file of compound 8

File Name: Peer Review File
File Description:

Supplementary Methods

General experimental information. All reagents were purchased from commercial sources (Sigma-Aldrich, Alfa Aesar, Spectrochem, Loba-biochem) and was used without further purification. Dry solvents for reaction purposes were purchased from commercial sources (Chemlabs, Finar). Column chromatography was performed with commercial solvents using silica gel (100-200 mesh particle size). Reactions were monitored by thin layer chromatography (TLC) on silica gel 60 F_{254}. NMR spectra were recorded in DMSO-d_6. ^{1}H (400 MHz) and ^{13}C (100 MHz) NMR were obtained on Bruker AVHDN-400 NMR spectrometer using tetramethylsilane (TMS) as internal standard. HRMS data were obtained using "6540 UHD Accurate-Mass Q-TOF LC/MS system (Agilent Technologies, Santa Clara, CA, USA) equipped with Agilent 1290 UPLC system". IR data were recorded on Thermo-Fisher Scientific FTIR instrument (model-Nicolet iS5, serial number- ASB1200503).

General Synthetic Method. To synthesize compounds **1-8**, an acyl hydrazide was coupled with an acid halide in presence of K_2CO_3 in DCM. To a stirred solution of an acyl hydrazide (1 equiv.) and K_2CO_3 (1.5 equiv.) in dichloromethane (DCM) was added an acid halide (1.3 equiv.) at 0°C. The reaction mixture was slowly allowed to come to room temperature and stirred for 4 hrs. The reaction mixture was then poured into cold water and organic layer was extracted with DCM (3 times). The organic extracts were dried over Na_2SO_4 and then evaporated under reduced pressure. The compounds were purified by column chromatography using hexane-ethyl acetate as eluent.

Synthetic routes for the synthesis of compounds 1-8

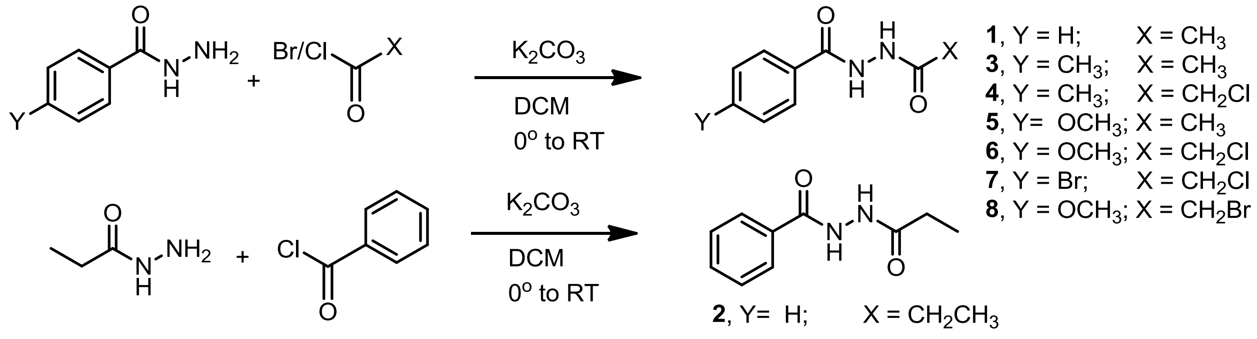

Compound **1**

Compound **1** was synthesized by coupling benzoic hydrazide (1.47 mmol) and acetyl chloride (1.91 mmol). After column chromatographic purification **1** was isolated in 88% yield as a white solid. ^{1}H NMR (400 MHz, DMSO): δ 10.26 (s, 1H), 9.87 (s, 1H), 7.87 (d, J = 8Hz, 2H), 7.57 (t, J = 8Hz, 1H), 7.49 (t, J=8Hz, 2H), 1.92(s, 3H); ^{13}C NMR (100MHz, DMSO): δ 168.5, 165.5, 132.5, 131.7, 128.4, 127.4, 20.6; HRMS: $[M+H]^+$ calcd. for $C_9H_{11}N_2O_2$, 179.0815; found, 179.0810; IR (Neat) (cm^{-1}): 3176, 3005, 1698, 1638, 1576.

Compound **2**

Compound **2** was synthesized by coupling propionic hydrazide (2.27 mmol) with benzoyl chloride (2.95 mmol). After column chromatographic purification **2** was isolated in 89% yield as a white solid. ^{1}H NMR (400 MHz, DMSO): δ 10.26 (s, 1H), 9.82 (s, 1H), 7.87 (d, J = 8Hz, 2H), 7.57 (t, J = 8Hz, 1H), 7.49 (t, J = 8Hz, 2H), 2.20 (q, J = 8Hz, 2H), 1.07 (t, J = 8Hz, 3H).; ^{13}C NMR (100 MHz, DMSO): δ 172.3, 165.5, 132.6, 131.7, 128.4, 127.4, 26.5, 9.6; HRMS: $[M+H]^+$ calcd. for $C_{10}H_{13}N_2O_2$, 193.0900; found, 193.0990]; IR (Neat) (cm^{-1}): 3287, 3223, 3033, 1698, 1651, 1628.

Compound **3**

Compound **3** was synthesized by coupling 4-methylbenzohydrazide (1.33 mmol) and acetyl chloride (1.73 mmol). After column chromatographic purification **3** was isolated in 94% yield as a white solid. ^{1}H NMR (400 MHz, DMSO): δ 10.18 (s,1H), 9.82 (s,1H), 7.77 (d, J = 8Hz 2H), 7.29 (d, J = 8Hz 2H), 2.36 (s,3H), 2.36 (s, 3H), 1.91 (s, 3H); ^{13}C NMR (100 MHz, DMSO): δ 168.5, 165.3, 141.7, 129.7, 128.9, 127.4, 21.0, 20.6; HRMS: $[M+Na]^+$ calcd. for $C_{10}H_{12}N_2NaO_2$, 215.0791; found, 215.0792; IR (Neat) (cm^{-1}): 3216, 3014, 1698, 1640, 1609.

Compound **4**

Compound **4** was synthesized by coupling 4-methylbenzohydrazide (1.33 mmol) and 2-chloroacetyl chloride (1.73 mmol). After column chromatographic purification **4** was isolated in 75% yield as a white solid. ^{1}H NMR (400 MHz, DMSO): δ 10.45 (s, 1H), 10.34 (s, 1H), 7.78 (d, J = 8Hz, 2H), 7.31 (d, J = 8Hz, 2H), 4.20 (s, 2H), 2.36 (s, 3H); ^{13}C NMR (100 MHz, DMSO): δ 165.4, 165.3, 142.0, 129.4, 129.0, 127.5, 41.0, 21.0; HRMS: $[M+H]^+$ calcd. for $C_{10}H_{11}ClN_2NaO_2$, 249.0401; found, 249.0401; IR (Neat) (cm^{-1}): 3265, 3183, 3028, 2922, 1682, 1651, 1603, 745.

Compound **5**

Compound **5** was synthesized by coupling 4-methoxybenzohydrazide (1.20 mmol) and acetyl chloride (1.50 mmol). After column chromatographic purification **5** was isolated in 92% yield as a white solid. ^{1}H NMR (400 MHz, DMSO): δ 10.13 (s, 1H), 9.82 (s,1H), 7.85 (d, J = 8Hz 2H), 7.02 (d, J = 8Hz 2H), 3.81 (s, 3H), 1.90 (s, 3H); ^{13}C NMR (100 MHz, DMSO): δ 168.6, 165.0, 162.0, 129.3, 124.7, 113.7, 55.4, 20.7; HRMS: $[M+H]^+$ calcd. for $C_{10}H_{13}N_2O_3$, 209.0921; found, 209.0925; IR (Neat) (cm^{-1}): 3251, 3003, 2933, 1683, 1598, 1569, 755.

Compound **6**

Compound **6** was synthesized by coupling 4-methoxybenzohydrazide (1.21 mmol) and 2-chloroacetyl chloride (1.56 mmol). After column chromatographic purification **6** was isolated in 79% yield as a white solid. ^{1}H NMR (400 MHz, DMSO): δ 10.38 (s, 1H), 10.32 (s, 1H), 7.86 (d, J = 8Hz, 2H), 7.03 (d, J = 8Hz, 2H), 4.19 (s, 2H), 3.82 (s, 3H); ^{13}C NMR (100MHz, DMSO): δ 165.5, 164.9, 162.1, 129.4, 124.3, 113.8, 55.4, 41.0; HRMS: $[M+Na]^+$ calcd. for $C_{10}H_{11}NClN_2NaO_3$, 265.0350; found, 265.0350; IR (Neat) (cm^{-1}): 3250, 1685, 1652, 1604, 779.

Compound **7**

Compound **7** was synthesized by coupling 4-bromobenzohydrazide (0.93 mmol) and 2-chloroacetyl chloride (1.21 mmol). After column chromatographic purification 7 was isolated in 75% yield as a white solid. ^{1}H NMR (400 MHz, DMSO): δ 10.63 (s, 1H), 10.42 (s, 1H), 7.81 (d, J = 8Hz, 2H), 7.73 (d, J = 8Hz, 2H), 4.20 (s, 2H); ^{13}C NMR (100MHz, DMSO): δ 165.4, 164.5, 131.6, 131.3, 129.6, 125.8, 41.0; HRMS: $[M+H]^+$ calcd. for $C_9H_9BrClN_2O_2$, 292.9508; found, 292.9506; IR (Neat) (cm^{-1}): 3171, 3007, 2921, 1677, 1587, 1562, 754.

Compound **8**

Compound **8** was synthesized by coupling 4-methoxybenzohydrazide (1.21 mmol) and 2-bromoacetyl bromide (1.56). After doing column chromatography **8** was isolated in 74% as white solid. ^{1}H NMR (400 MHz, DMSO) δ 10.37 (s, 1H), 10.34 (s, 1H), 7.86 (d, J= 8Hz, 2H), 7.03 (d, J = 8Hz, 2H), 3.98 (s, 2H), 3.82 (s, 3H); ^{13}C NMR (100 MHz, DMSO) δ 165.5, 164.8, 162.1, 129.4, 124.3, 113.7, 55.4, 27.2; HRMS: $[M+Na]^+$ calcd. for $C_{10}H_{10}BrN_2NaO_3$, 308.9845; found 308.9870; IR (Neat) (cm^{-1}): 3207, 3039, 1685, 1632, 1600, 769.

Supplementary Table 1. Details of crystallization conditions of compounds **1-8**.

**Comp**	**Crystallization Solvent**	**Temperature**
**1**	Dichloromethane : Hexane (3:1)	4ºC
**2**	Dichloromethane : Hexane (3:1)	4ºC
**3**	Ethyl acetate: Hexane (3:1)	RT
**4**	Acetonitrile	RT
**5**	Dichloromethane (This compound crystallized out from reaction mixture before column purification)	RT
**6**	Ethyl acetate: Hexane (3:1)	RT
**7**	Ethyl acetate	RT
**8**	Ethyl acetate: Hexane (3:1)	RT

Supplementary Table 2. Resolution of crystal structures of compounds **1-8**.

Comp	Resolution (Å)	Comp	Resolution (Å)	Comp	Resolution (Å)	Comp	Resolution (Å)
**1**	0.82	**3**	0.80	**5**	0.82	**7**	0.80
**2**	0.84	**4**	0.82	**6**	0.82	**8**	0.82

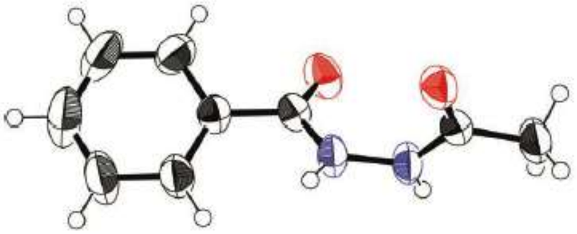

1 (CCDC 1486577)

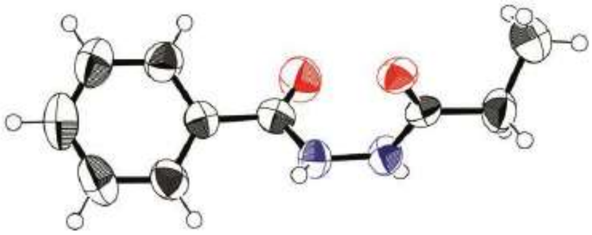

2 (CCDC 1486578)

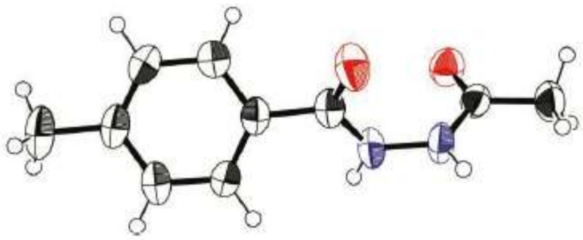

3 (CCDC 1486579)

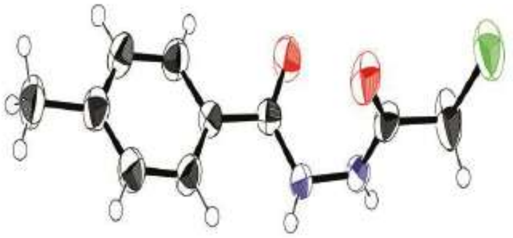

4 (CCDC 1486581)

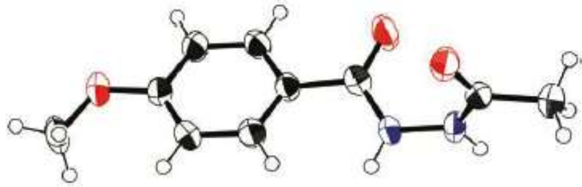

5 (CCDC 1486580)

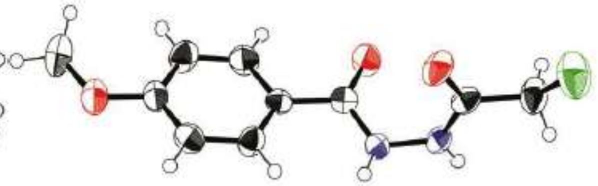

6 (CCDC 1486582)

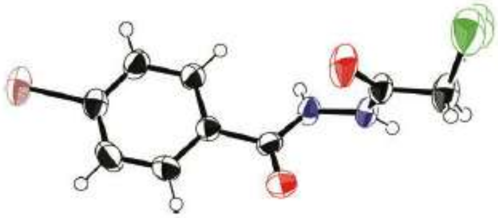

7 (CCDC 1486583)

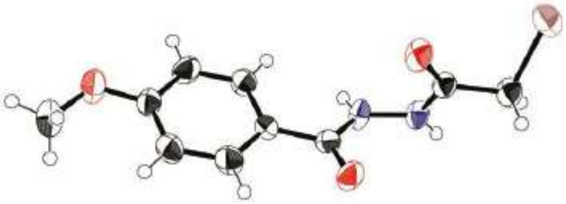

8 (CCDC 1486584)

Supplementary Figure 1. ORTEP diagram of crystal structures of compounds **1-8**.

Supplementary Table 3. Structural and NBO data of compound **1-8**. NBO analyses were carried out by using B3LYP/6-311+G(2d,p) or HF/6-311+G(2d,p) level of theory. $E^1_{(n\to\pi^*)}$ and $E^2_{(n\to\pi^*)}$ were defined in the main manuscript. $E^1_{(\pi\to\pi^*)}$ is the NBO second order perturbation energy for electron donation from filled π orbital of C=O bond of the first carbonyl (CO-I) to the antibonding π^* orbital of C=O bond of the second carbonyl (CO-II). $E^2_{(\pi\to\pi^*)}$ is the NBO second order perturbation energy for electron donation from filled π orbital of C=O bond of the second carbonyl (CO-II) to the antibonding π^* orbital of C=O bond of the first carbonyl (CO-I). $E^t_{(\pi\to\pi^*)} = E^1_{(\pi\to\pi^*)} + E^2_{(\pi\to\pi^*)}$; $E^t = E^t_{(n\to\pi^*)} + E^t_{(\pi\to\pi^*)} = E^1_{(n\to\pi^*)} + E^2_{(n\to\pi^*)} + E^1_{(\pi\to\pi^*)} + E^2_{(\pi\to\pi^*)}$; $E^t_{(n\to\pi^*)}$ values are taken from Table 1 of the manuscript. [CO-I and CO-II are defined in Fig. 1d of the main paper]. NP = Not present

Comp	Y	X	$n\to\pi^*$ (kcal.mol^{-1})		$\pi\to\pi^*$ (kcal.mol^{-1})		$\pi\to\pi^*$ (kcal.mol^{-1})		Total $\pi\to\pi^*$ (kcal.mol^{-1})	Total stabilization energy (kcal.mol^{-1})
			HF/6-311+G(2d,p)		HF/6-311+G(2d,p)		B3LYP/6-311+G(2d,p)		B3LYP/6-311+G(2d,p)	B3LYP/6-311+G(2d,p)
			$E^1_{(n\to\pi^*)}$	$E^2_{(n\to\pi^*)}$	$E^1_{(\pi\to\pi^*)}$	$E^2_{(\pi\to\pi^*)}$	$E^1_{(\pi\to\pi^*)}$	$E^2_{(\pi\to\pi^*)}$	$E^t_{(\pi\to\pi^*)}$	E^t
**1**	H	CH_3	NP	NP	0.02	0.02	0.01	0.01	0.02	**0.02**
**2**	H	CH_2CH_3	0.06	0.05	0.02	0.02	0.01	0.01	0.02	**0.08**
**3**	CH_3	CH_3	0.02	0.02	0.02	0.02	0.02	0.01	0.03	**0.06**
**4**	CH_3	CH_2Cl	0.15	0.46	0.03	0.04	0.02	0.02	0.04	**0.48**
**5**	OCH_3	CH_3	0.05	0.23	NP	0.01	NP	NP	NP	**0.19**
**6**	OCH_3	CH_2Cl	0.19	0.57	0.03	0.03	0.01	0.02	0.03	**0.59**
**7**	Br	CH_2Cl	0.12	0.52	0.02	0.05	0.01	0.03	0.04	**0.51**
**8**	OCH_3	CH_2Br	0.18	0.54	0.03	0.04	0.02	0.02	0.04	**0.58**

Supplementary Table 4. Electron donation from the halogen (Cl or Br) lone pairs to the antibonding π* and σ* orbitals of nearby carbonyl C=O bonds in compounds **4**, **6-8**. The calculations are carried out at B3LYP/6-311+G(2d,p) level of theory. X-C-C=O torsion angles are from crystal geometries.

comp	X-C-C=O torsion angle (X = Cl or Br)	$E_{(n\to\pi^*)}$	$E_{(n\to\sigma^*)}$	$E^t = E_{(n\to\pi^*)} + E_{(n\to\sigma^*)}$
		kcal.mol^{-1}	kcal.mol^{-1}	kcal.mol^{-1}
**4**	-1.2	0.23	0.44	0.67
**6**	33.3	0.26	0.43	0.69
**7**	-61.6	1.06	0.30	1.36
**8**	-35.8	0.19	0.44	0.63

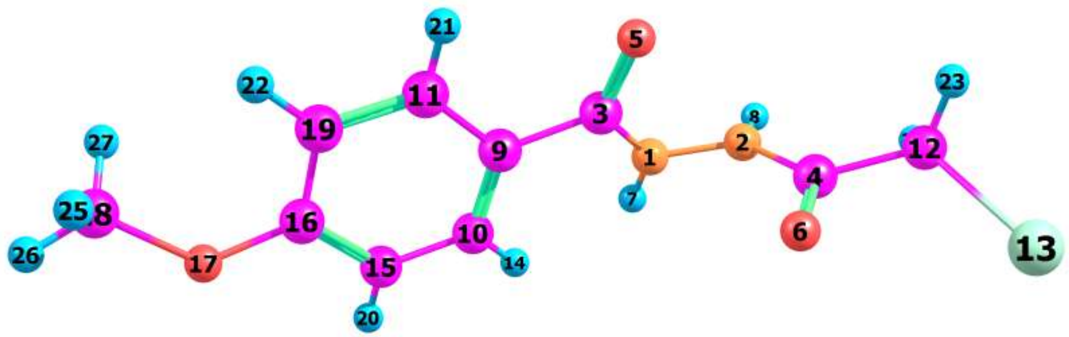

Supplementary Figure 2. A representative molecular geometry used for geometry optimization. The geometry used for compound **6** is shown here. The dihedral angles that were kept frozen during optimizations are between atoms 4-2-1-3, 5-3-1-2 and 6-4-2-1 shown in the Figure. This Figure is generated by using Chemcraft software.

Supplementary Table 5. B3LYP/6-311+G(2d,p) level optimized structural parameters and NBO data for compounds **1-8**. The dihedral angles involved in the twisted conformation of the two carbonyl groups were kept frozen to their X-ray values (see Supplementary Figure 4 below). $d_1 = O^1 \cdots C^2$; $d_2 = O^2 \cdots C^1$; $\theta_1 = \angle O^1 \cdots C^2{=}O^2$; $\theta_2 = \angle O^2 \cdots C^1{=}O^1$ (Fig. 1d). $E^1_{(n\rightarrow\pi^*)}$ and $E^2_{(n\rightarrow\pi^*)}$ are defined in Table 1. $E^t_{(n\rightarrow\pi^*)} = E^1_{(n\rightarrow\pi^*)} + E^2_{(n\rightarrow\pi^*)}$. NP = Not present

**Comp**	**Y**	**X**	**d_1**	**d_2**	**θ_1**	**θ_2**	***n→π****		**Total *n→π****
			**(Å)**	**(Å)**	**(°)**	**(°)**	**(kcal.mol^{-1})**		**(kcal.mol^{-1})**
							$E^1_{(n\rightarrow\pi^*)}$	$E^2_{(n\rightarrow\pi^*)}$	$E^1_{(n\rightarrow\pi^*)} + E^2_{(n\rightarrow\pi^*)}$
**1**	H	CH_3	3.695	3.667	100.6	102.4	NP	NP	**NP**
**2**	H	CH_2CH_3	3.340	3.362	94.3	93.2	0.03	0.03	**0.06**
**3**	CH_3	CH_3	3.386	3.411	93.3	92.1	0.02	0.02	**0.04**
**4**	CH_3	CH_2Cl	3.182	3.149	84.2	85.7	0.06	0.24	**0.30**
**5**	OCH3	CH_3	3.271	3.219	82.6	84.9	0.02	0.12	**0.14**
**6**	OCH_3	CH_2Cl	3.122	3.102	83.6	84.5	0.09	0.26	**0.35**
**7**	Br	CH_2Cl	3.153	3.118	83.4	85.1	0.05	0.23	**0.28**
**8**	OCH_3	CH_2Br	3.117	3.105	83.6	84.3	0.05	0.24	**0.29**

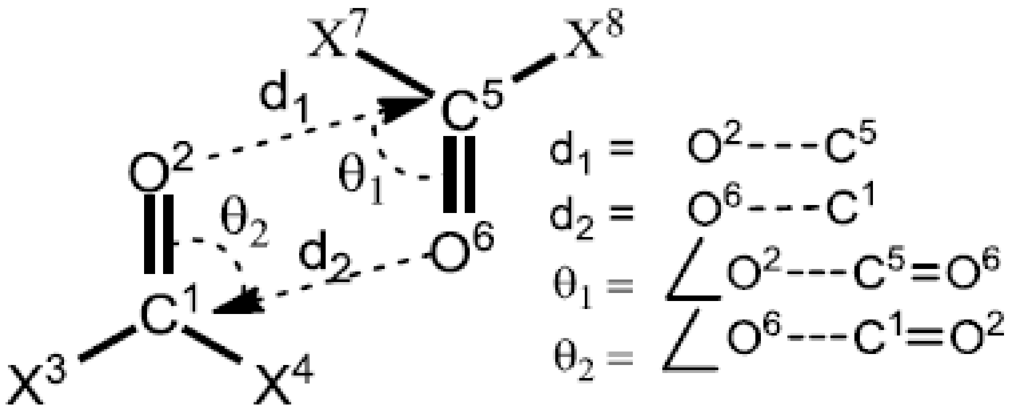

Supplementary Figure 3. Fragment used to search intramolecular reciprocal C=O···C=O short contacts.

Supplementary Table 6. Compounds obtained from CSD search with various types of C=O···C=O short contacts.

Number of bonds between carbonyl groups	Interaction Type	Number of compounds
3	1,5	1212
4	1,6	163
5	1,7	38
6	1,8	15
7	1,9	3
14	1,16	1
	Total	1432

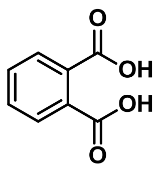
PHTHAC01

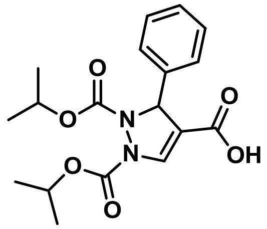
PODHUM

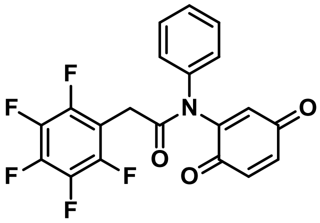
GECYEU

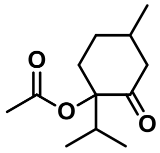
LEBRER

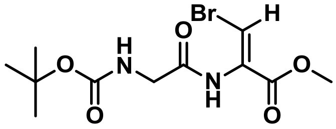
KOXBIK

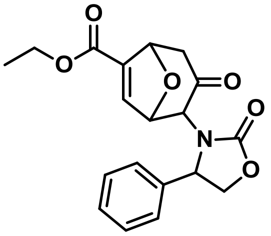
CAJVIU

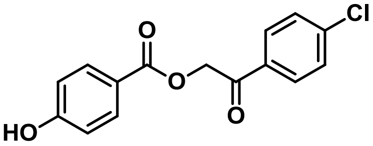
AZULUD

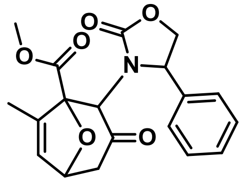
ZUKVUY

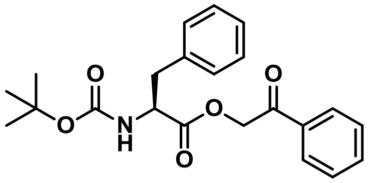
GAPDIK

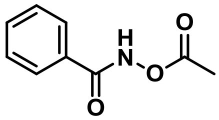
LAGTIX

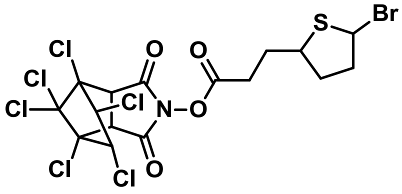
SUDAXAS01

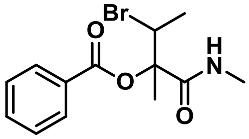
JUHQEK

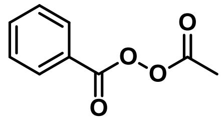
ACBZO01

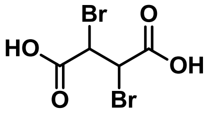
WOCHIF

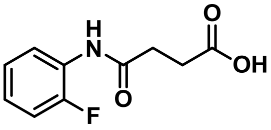
MODYIO

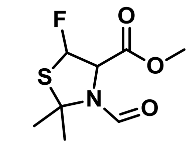

YEXQOH

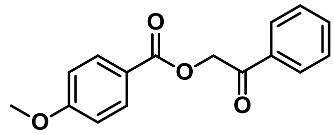

CIQNEW

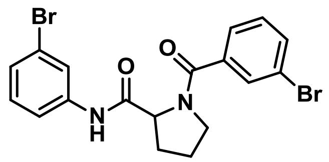

LUCHEY

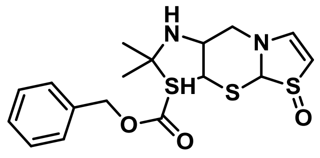

BECLAW

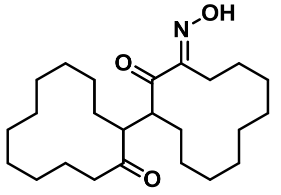

DESPAT

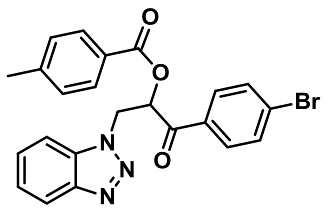

GIRQAA

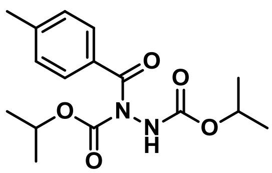

PUFBEZ

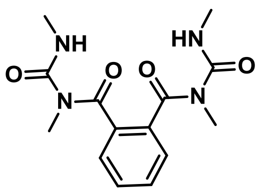

JOSGIH

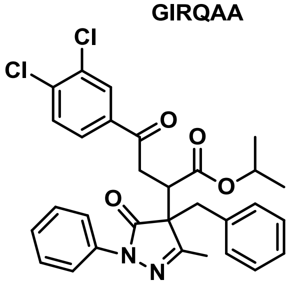

IQAXII

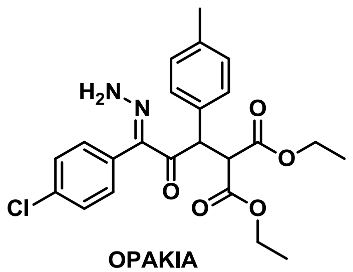

OPAKIA

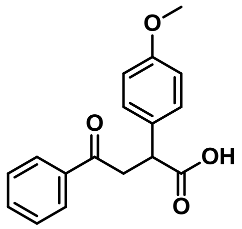

OMINII

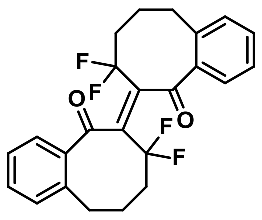

XACLUK

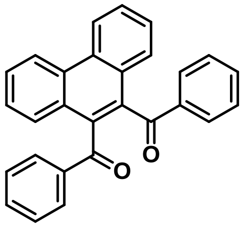

EZELOK01

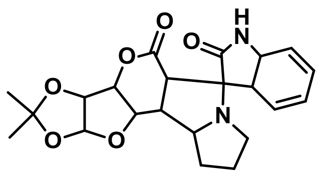

WIHKAB

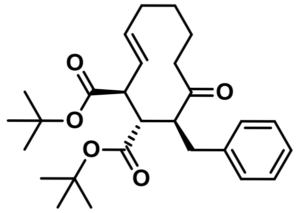

LOVNIT

Supplementary Figure 4. 30 compounds from the CSD search that were chosen for NBO analysis.

Supplementary Table 7. Structural and NBO data of 30 randomly selected molecules from the CSD with various d_1 and d_2 values. The molecules are selected in such a way that the whole range of ∠O···C=O angle θ is covered. NBO analyses was carried out by using B3LYP/6-311+G(2d,p) or HF/6-311+G(2d,p) level of theory. $E^1_{(n\rightarrow\pi^*)}$ and $E^2_{(n\rightarrow\pi^*)}$ were defined in the main manuscript. $E^1_{(\pi\rightarrow\pi^*)}$ and $E^2_{(\pi\rightarrow\pi^*)}$ are defined in Supplementary Table 3. $E^t_{(\pi\rightarrow\pi^*)} = E^1_{(\pi\rightarrow\pi^*)} + E^2_{(\pi\rightarrow\pi^*)}$; $E^t = E^t_{(n\rightarrow\pi^*)} + E^t_{(\pi\rightarrow\pi^*)} = E^1_{(n\rightarrow\pi^*)} + E^2_{(n\rightarrow\pi^*)} + E^1_{(\pi\rightarrow\pi^*)} + E^2_{(\pi\rightarrow\pi^*)}$; $E^t_{(n\rightarrow\pi^*)}$ values are taken from Table 2 of the manuscript. [CO-I and CO-II are randomly chosen in these molecules]. NP = Not present

CCDC Ref. code	n→π* (kcal.mol^{-1})		π→π* (kcal.mol^{-1})		π→π* (kcal.mol^{-1})		Total π→π* (kcal.mol^{-1})	Total stabilization energy (kcal.mol^{-1})
	HF/6-311+G(2d,p)		HF/6-311+G(2d,p)		B3LYP/6-311+G(2d,p)		B3LYP/6-311+G(2d,p)	B3LYP/6-311+G(2d,p)
	$E^1_{(n\rightarrow\pi^*)}$	$E^2_{(n\rightarrow\pi^*)}$	$E^1_{(\pi\rightarrow\pi^*)}$	$E^2_{(\pi\rightarrow\pi^*)}$	$E^1_{(\pi\rightarrow\pi^*)}$	$E^2_{(\pi\rightarrow\pi^*)}$	$E^t_{(\pi\rightarrow\pi^*)}$	E^t
PHTHAC05	0.20	0.20	NP	NP	0.02	0.02	0.04	**0.29**
PODHUM	0.10	0.10	NP	NP	NP	NP	NP	**0.11**
GECYEU	0.46	0.23	NP	NP	NP	NP	NP	**0.47**
LEBRER	1.04	0.80	0.02	NP	NP	NP	NP	**1.37**
KOXBIK	0.45	0.20	NP	NP	NP	NP	NP	**0.44**
CAJVIU	0.57	0.18	0.03	0.04	0.01	0.03	0.04	**0.57**
AZULUD	0.52	0.29	0.01	0.02	NP	NP	NP	**0.56**
ZUKVUY	1.09	0.58	0.18	0.26	0.09	0.16	0.25	**1.45**
GAPDIK	0.10	0.30	0.04	NP	0.02	0.02	0.04	**0.33**
LAGTIX	0.24	0.10	NP	NP	NP	NP	NP	**0.24**
SUDAXAS01	0.27	0.12	NP	0.03	NP	0.02	0.02	**0.31**
JUHQEK	1.21	1.21	0.19	0.25	0.10	0.13	0.23	**2.06**
ACBZO01	0.28	0.34	0.03	0.03	0.01	0.01	0.02	**0.5**
WOCHIF	0.72	1.09	0.28	0.37	0.14	0.20	0.34	**1.74**
MODYIO	0.22	0.22	0.06	0.06	0.03	0.03	0.06	**0.38**
YEXQOH	0.10	0.21	0.01	NP	NP	NP	NP	**0.21**
CIQNEW	0.32	0.30	0.06	0.03	0.03	0.01	0.04	**0.47**
LUCHEY	0.72	0.33	0.11	0.11	0.06	0.07	0.13	**0.94**
BECLAW	1.43	0.75	0.47	0.16	0.28	0.09	0.37	**2.07**
DESPAT	0.25	1.24	0.24	0.60	0.14	0.39	0.53	**1.7**
GIRQAA	0.45	0.47	0.28	0.22	0.17	0.13	0.30	**0.98**
PUFBEZ	0.10	0.27	0.07	0.06	0.06	0.06	0.12	**0.4**
JOSGIH	0.36	0.40	0.85	0.43	NP	0.22	0.22	**0.78**
IQAXII	0.42	0.27	0.53	0.44	0.32	0.26	0.58	**1.06**
OPAKIA	0.29	0.44	0.48	0.54	0.31	0.33	0.64	**1.21**
OMINII	0.40	0.27	0.39	0.25	0.23	0.14	0.37	**0.9**
XACLUK	0.30	0.38	1.27	1.42	1.27	1.42	2.69	**3.37**
EZELOK01	0.19	0.13	0.91	0.59	0.63	0.40	1.03	**1.31**
WIHKAB	0.71	0.07	0.85	0.69	0.55	0.42	0.97	**1.58**
LOVNIT	0.24	0.05	1.02	0.72	0.69	0.54	1.23	**1.5**

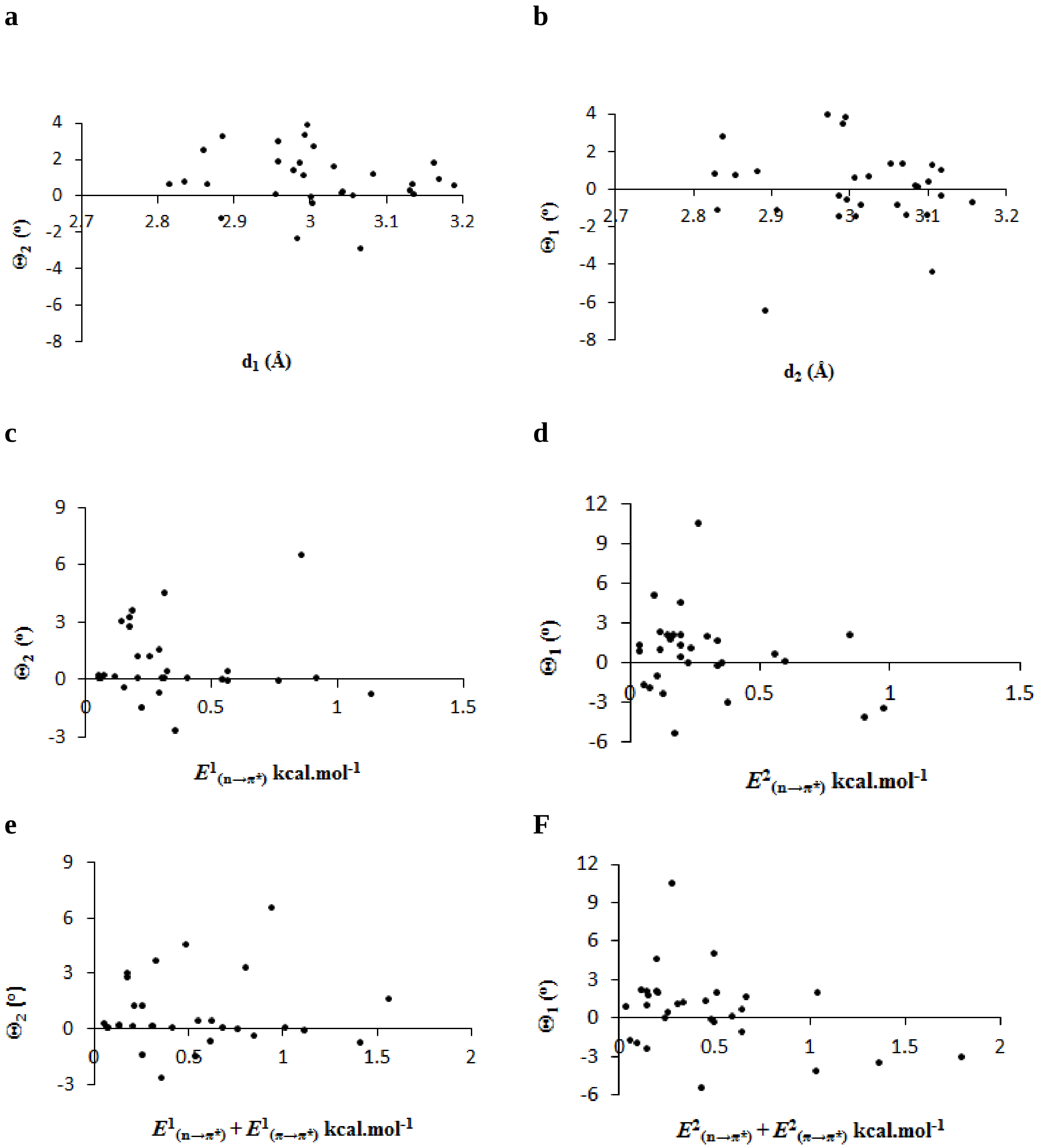

Supplementary Figure 5. Plots of pyramidality (Θ) of acceptor carbonyl carbon atoms. The pyramidality (Θ) were plotted against O···C (d_1 and d_2) distances and C=O···C=O interaction energies in the molecules obtained from the CSD search shown in Table 2. **a**, Θ_2 vs. d_1. **b**, Θ_1 vs. d_2. **c**, Θ_2 vs. $E^1_{(n\rightarrow\pi^*)}$. **d**, Θ_1 vs. $E^2_{(n\rightarrow\pi^*)}$. **e**, Θ_2 vs. [$E^1_{(n\rightarrow\pi^*)}$ + $E^1_{(\pi\rightarrow\pi^*)}$].**f**, Θ_1 vs. [$E^2_{(n\rightarrow\pi^*)}$ + $E^2_{(\pi\rightarrow\pi^*)}$]. d_1, d_2, Θ_1, Θ_2, $E^1_{(n\rightarrow\pi^*)}$ and $E^2_{(n\rightarrow\pi^*)}$ values are taken from Table 2. $E^1_{(\pi\rightarrow\pi^*)}$ and $E^2_{(\pi\rightarrow\pi^*)}$ values are taken from Supplementary Table 7.

Supplementary Table 8. Analysis of 1, 5-reciprocal interactions from the CSD with both d_1 and d_2 ≤ 3.00 Å. A total of 249 molecules were obtained that have 1, 5-reciprocal C=O···C=O interactions with both d_1 and $d_2 \leq 3.00$ Å.

Groups Between the two C=O group	Number of molecules	Percentage (%)
One heteroatom (O or N), one chiral C atom	117	46.98
Both are chiral C atoms	61	24.49
One heteroatom (O or N), one achiral C atom	28	11.24
Both sp^2 C atoms [the C=O groups are attached to 1,2-positions of benzene, naphthalene or non-aromatic rings]	21	8.43
Both are heteroatoms (O or N)	14	5.62
One chiral and one achiral C atoms	4	1.60
Both are achiral C atoms [attached to rings with conformational constraints]	4	1.60

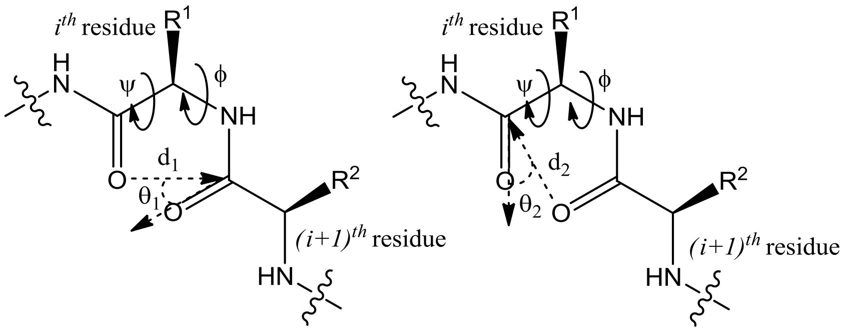

Supplementary Figure 6. Definition of various structural parameters (d_1, d_2, θ_1, θ_2, ϕ and ψ) used for the PDB search and generating the Ramachandran plots.

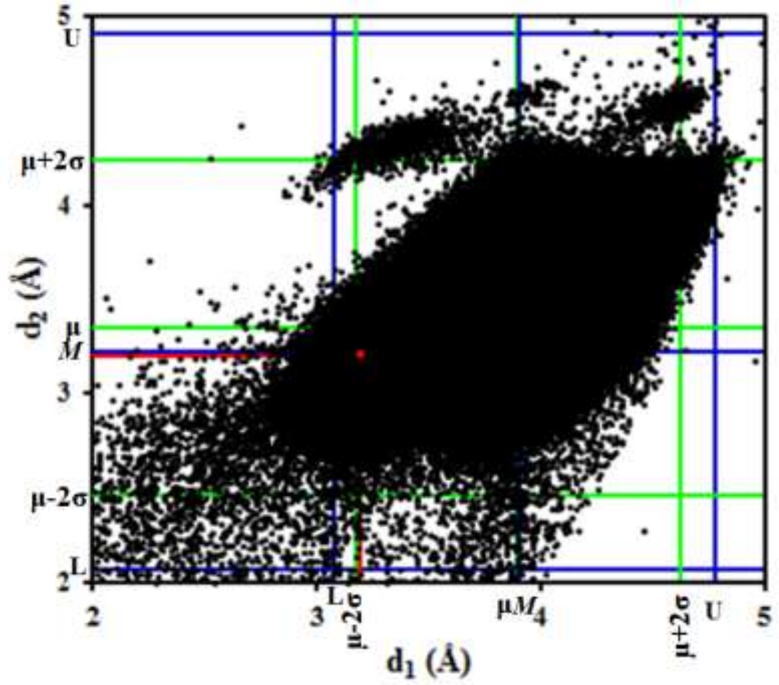

Supplementary Figure 7. Plot showing the distribution of O···C distances (d_1 and d_2) in all amino acids in 2184 proteins studied. We chose all the chains present in each of the protein for our analysis. The red box with the red point at (3.2, 3.2) indicates reciprocal interactions (d_1 and $d_2 \leq 3.2$ Å). Two standard deviation (SD) analyses are presented by green lines. d_1 and d_2 values lower than (μ-2σ) and higher than (μ+2σ) represents outliers [μ =mean; σ = standard deviation). Outlier labeling rule analyses using 2.2 as multiplier are presented by blue lines. *M* is the median and d_1 and d_2 values lower than L and higher than U represents outliers.

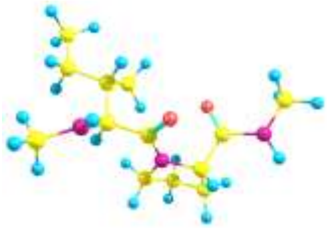
Ile-Pro (135-136) PDB: 2opc

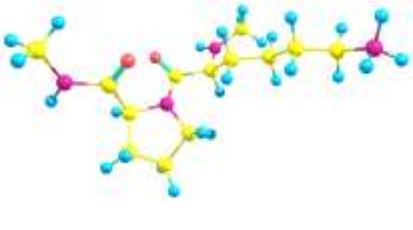
Lys-Pro (50-51) PDB: 1k3i

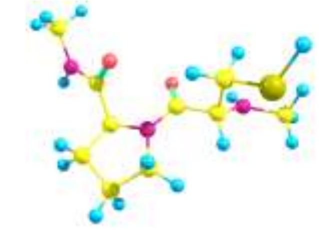
Cys-Pro (251-252) PDB: 1gcy

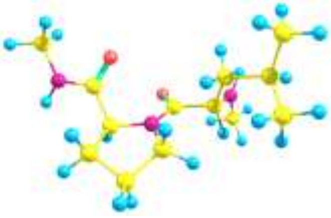
Leu-Pro (379-380) PDB: 1g5a

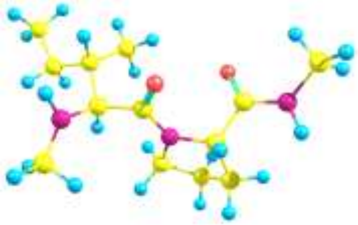
Ile-Pro (107-108) PDB: 1o7i

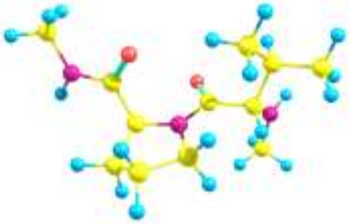
Val-Pro (294-295) PDB: 1jnd

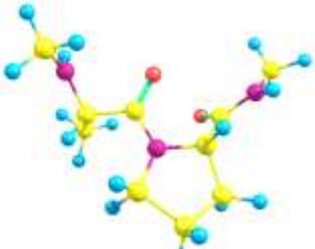
Ala-Pro (264-265) PDB: 2xu9

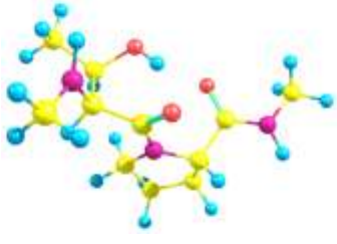
Thr-Pro (3-4) PDB: 1fj2

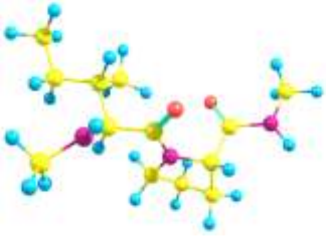
Ile-Pro (208-209) PDB: 1e2w

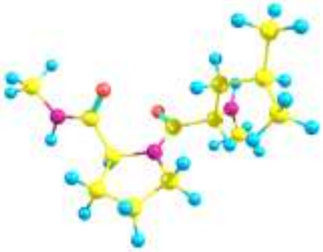
Leu-Pro (20-21) PDB: 1a2p

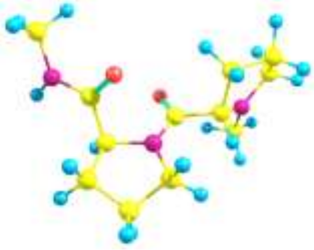
Pro-Pro (186-187) PDB: 3cx2

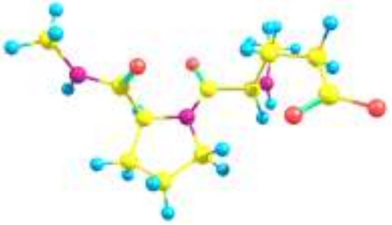
Glu-Pro (623-624) PDB: 1eu1

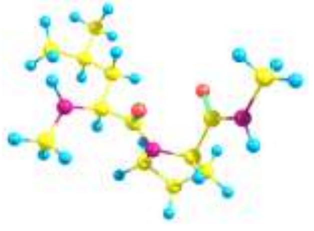
Leu-Pro (141-142) PDB: 2x5o

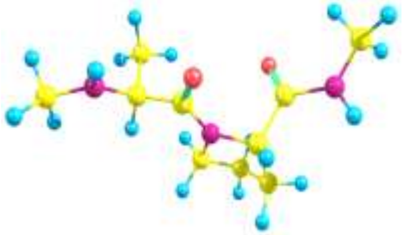
Ala-Pro (103-104) PDB: 1g12

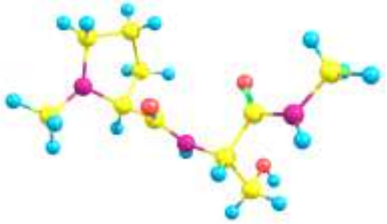
Pro-Ser (32-33) PDB: 4psc

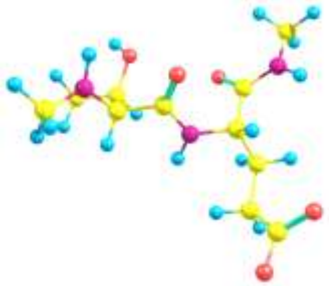
Thr-Glu (108-109) PDB: 4pdy

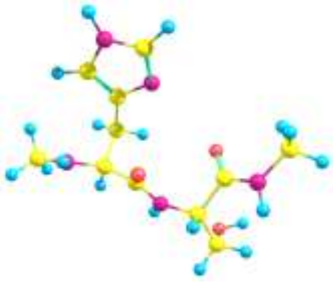
His-Ser (331-332) PDB: 1b6a

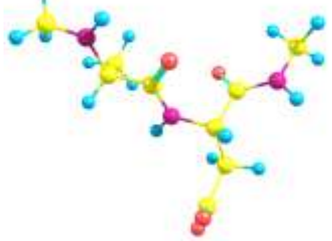
Ala-Asp (95-96) PDB: 2bi8

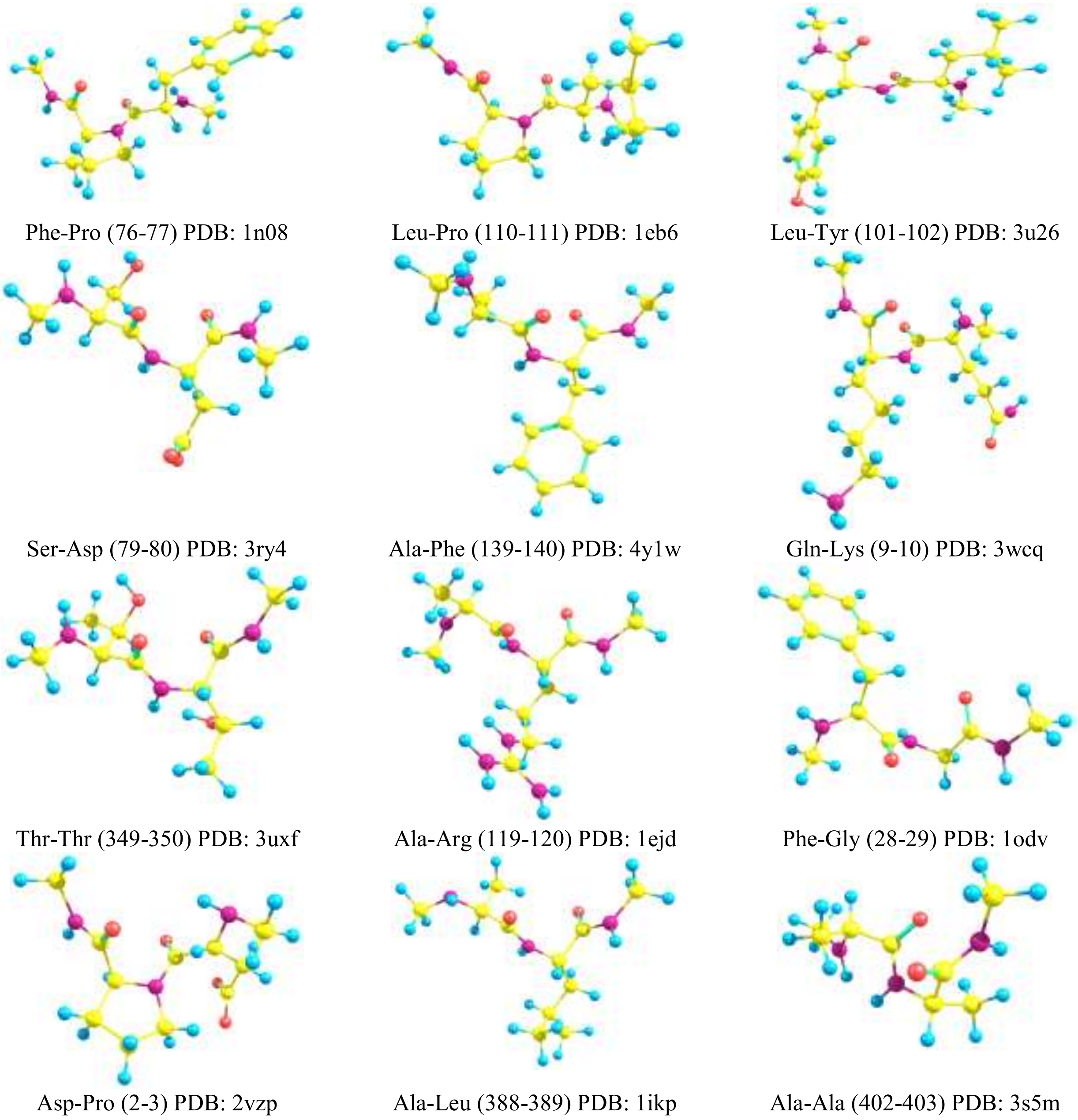

Supplementary Figure 8. Amino acid pairs having reciprocal C═O···C═O interactions chosen for NBO analysis.

Supplementary Table 9. Structural and NBO data of 30 randomly selected amino acid pairs from the PDB with various d_1 and d_2 values ≤ 3.2 Å. The amino acid pairs are selected in such a way that the whole range of ∠O···C=O angle θ is covered. NBO analyses was carried out by using B3LYP/6-311+G(2d,p) or HF/6-311+G(2d,p) level of theory. $E^1_{(n\rightarrow\pi^*)}$, $E^2_{(n\rightarrow\pi^*)}$ and $E^t_{(n\rightarrow\pi^*)}$ were defined in the main manuscript. $E^1_{(\pi\rightarrow\pi^*)}$ and $E^2_{(\pi\rightarrow\pi^*)}$ are defined in Supplementary Table 6. $E^t_{(\pi\rightarrow\pi^*)} = E^1_{(\pi\rightarrow\pi^*)} + E^2_{(\pi\rightarrow\pi^*)}$; $E^t = E^t_{(n\rightarrow\pi^*)} + E^t_{(\pi\rightarrow\pi^*)} = E^1_{(n\rightarrow\pi^*)} + E^2_{(n\rightarrow\pi^*)} + E^1_{(\pi\rightarrow\pi^*)} + E^2_{(\pi\rightarrow\pi^*)}$; $E^t_{(n\rightarrow\pi^*)}$ values are taken from Table 3 of the manuscript. [CO-I and CO-II are randomly chosen]. NP = Not present

**Amino acid pair**	**PDB Code**	**Residue Number**	***n→π**** **(kcal.mol⁻¹)**		***π→π**** **(kcal.mol⁻¹)**		***π→π**** **(kcal.mol⁻¹)**		**Total *π→π**** **(kcal.mol⁻¹)**	**Total stabilization Energy (kcal.mol⁻¹)**
			HF/6-311+G(2d,p)		HF/6-311+G(2d,p)		B3LYP/6-311+G(2d,p)		B3LYP/6-311+G(2d,p)	B3LYP/6-311+G(2d,p)
			$E^1_{(n\rightarrow\pi^*)}$	$E^2_{(n\rightarrow\pi^*)}$	$E^1_{(\pi\rightarrow\pi^*)}$	$E^2_{(\pi\rightarrow\pi^*)}$	$E^1_{(\pi\rightarrow\pi^*)}$	$E^2_{(\pi\rightarrow\pi^*)}$	$E^t_{(\pi\rightarrow\pi^*)}$	E^t
Ile-Pro	2opc	135-136	2.34	0.66	0.14	0.11	NP	NP	NP	**2.49**
Lys-Pro	1k3i	50-51	0.60	0.57	NP	NP	NP	NP	NP	**0.82**
Cys-Pro	1gcy	251-252	1.62	0.80	0.09	0.12	0.03	0.04	0.07	**1.91**
Leu-Pro	1g5a	379-380	0.79	0.35	0.05	0.07	0.02	0.02	0.04	**0.92**
Ile-Pro	1o7i	107-108	0.61	0.31	NP	0.02	0.03	NP	0.03	**0.66**
Val-Pro	1jnd	294-295	0.25	0.14	NP	0.04	NP	NP	NP	**0.27**
Ala-Pro	2xu9	264-265	4.06	0.74	0.26	0.06	0.19	0.03	0.22	**4.41**
Thr-Pro	1fj2	3-4	0.36	0.46	0.06	NP	0.03	NP	0.03	**0.63**
Ile-Pro	1e2w	208-209	0.34	0.12	NP	0.05	NP	NP	NP	**0.31**
Leu-Pro	1a2p	20-21	0.95	0.34	0.15	0.08	0.09	0.03	0.12	**1.17**
Pro-Pro	3cx2	186-187	1.26	0.36	0.20	0.02	0.18	0.24	0.42	**1.79**
Glu-Pro	1eu1	623-624	0.30	0.11	0.01	0.02	0.03	NP	0.03	**0.28**
Leu-Pro	2x5o	141-142	1.79	1.71	1.04	0.86	0.62	0.49	1.11	**3.99**
Ala-Pro	1g12	103-104	0.44	0.05	0.08	0.06	NP	NP	NP	**0.42**
Pro-Ser	4psc	32-33	0.59	0.43	0.25	0.25	0.15	0.15	0.30	**1.09**
Thr-Glu	4pdy	108-109	0.91	0.26	0.33	0.28	0.11	0.11	0.22	**1.19**
His-Ser	1b6a	331-332	0.20	0.15	0.15	0.17	0.09	0.11	0.30	**0.57**
Ala-Asp	2bi8	95-96	0.66	0.15	0.27	0.24	0.19	0.18	0.37	**1.02**
Phe-Pro	1n08	76-77	0.72	0.12	0.27	0.23	0.23	0.21	0.44	**1.06**
Leu-Pro	1eb6	110-111	0.19	0.14	0.02	0.05	0.08	0.04	0.12	**0.36**
Leu-Tyr	3u26	101-102	1.28	0.39	0.82	0.50	0.55	0.32	0.87	**2.17**
Ser-Asp	3ry4	79-80	0.79	0.14	0.33	0.23	0.17	0.12	0.29	**0.97**
Ala-Phe	4y1w	139-140	0.70	0.31	0.60	0.43	0.37	0.28	0.65	**1.45**
Gln-Lys	3wcq	9-10	1.25	0.22	1.04	0.94	0.65	0.66	1.31	**2.5**
Thr-Thr	3uxf	349-350	0.38	0.16	0.43	0.36	0.29	0.26	0.55	**0.97**
Ala-Arg	1ejd	119-120	0.19	NP	0.18	0.12	0.11	0.09	0.20	**0.33**
Phe-Gly	1odv	28-29	0.16	0.03	0.33	0.20	0.23	0.15	0.38	**0.53**
Asp-Pro	2vzp	2-3	0.12	0.26	1.02	0.84	0.70	0.59	1.29	**1.55**
Ala-Leu	1ikp	388-389	0.19	NP	0.27	0.11	0.19	0.10	0.29	**0.41**
Ala-Ala	3s5m	402-403	1.01	0.02	3.46	0.72	2.69	0.50	3.19	**4.09**

Supplementary Table 10. The 10 proteins with most number of reciprocal C=O···C=O interactions ranked by frequency. Their secondary structure assignment for regions having amino acid pairs involved in reciprocal C=O···C=O interactions show that coils and turns have predominant presence of reciprocal C=O···C=O interactions in these proteins.

PDB code	Rank	Instances of reciprocal C=O···C=O interaction	Percentage of amino acid residue involved in reciprocal interaction (%)	Secondary structure	manually assigned
4LGY	1	92	7.14	Coil	45
				Turn	10
				Strand	10
				α-helix	2
3OG2	2	85	8.62	Coil	26
				Turn	29
				Strand	4
				α-helix	1
4I3G	3	85	5.46	Coil	46
				Turn	6
				Strand	10
				α-helix	1
3BVU	4	84	8.28	Coil	42
				Turn	12
				Strand	8
				α-helix	1
4TZ1	5	80	0.58	Coil	31
				Turn	24
				Strand	6
3TEW	6	77	10.98	Coil	31
				Strand	9
				Turn	13
1IKP	7	76	12.69	Coil	35
				Turn	12
				Strand	7
3WEO	8	72	8.71	Coil	28
				Turn	10
				Strand	10
1K3I	9	69	10.599	Coil	29
				Turn	0
				Strand	0
4WFO	10	69	8.35	Coil	25
				Turn	15
				Strand	3
				α-helix	2

Supplementary Table 11. Distribution of reciprocal C=O···C=O interactions in various secondary structures in the 10 proteins with the most number of reciprocal C=O···C=O interactions.

**Type of Secondary structure**	**Number reciprocal C=O···C=O interactions**
Coil	207
β-turn	84
β-sheet	17
α-helix	10
β-sheet & Coil interface	46
α-Helix & Coil interface	40
β-sheet & α-Helix interface	6
β-turn & coil interface	2

Supplementary Table 12. Number of reciprocal C=O···C=O interactions found in various types of β-turns present in the proteins listed in Table 10.

**β-turn type**	**Number of reciprocal C=O···C=O interactions**
II	43
IV	26
II'	5
I	4
VIII	4
VIb	2

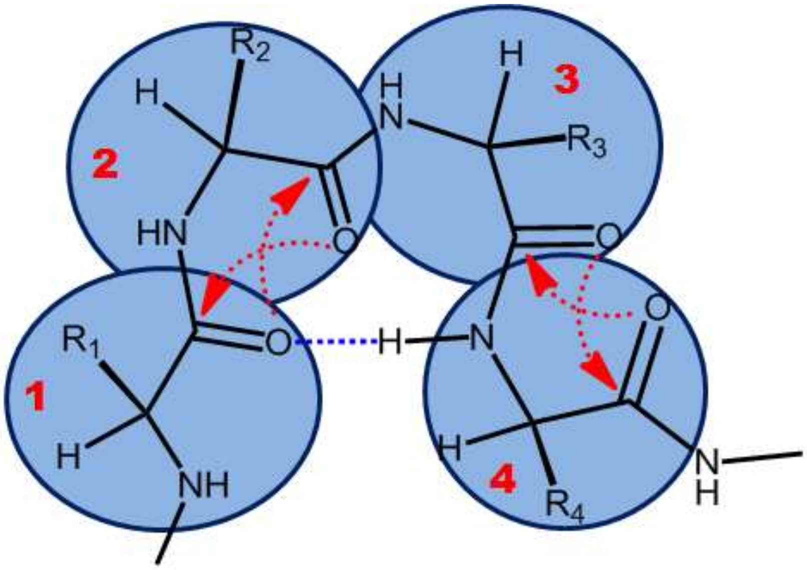

Supplementary Figure 9. Schematic diagram of a peptide β-turn. In β-turns, reciprocal C=O···C=O interactions are present between residue 1-2 and residue 3-4 (shown by red curved dotted arrow). Also the hydrogen bond between residues 1-4 in the turn is shown as blue dotted straight line.

Supplementary Table 13. NBO deletion analyses carried out on crystal geometries at HF/6-311+G(2d,p) level of theory. ΔE_{del} is the NBO deletion energy for the orbital interaction energy between the donor carbonyl oxygen lone pair (n_O) and the $\pi^*_{C=O}$ orbital of the acceptor carbonyl C=O bond. $\Delta q(\pi^*)$ is the increase in the charge of the $\pi^*_{C=O}$ orbital due to the orbital interaction with the donor carbonyl oxygen lone pair (n_O). $\Delta q(n_o)$ is the decrease in charge in the donor oxygen lone pair (n_O) due to the orbital interaction with the acceptor $\pi^*_{C=O}$ orbital.

Compound	ΔE_{del} (kcal.mol^{-1})	$\Delta q(\pi^*)$ (electron)		$\Delta q(n_O)$ (electron)	
		CO-I to CO-II	CO-II to CO-I	CO-I to CO-II	CO-II to CO-I
**1**	-	-	-	-	-
**2**	0.057	+0.00006	+0.00005	-0.00014	-0.00011
**3**	0.031	0.00000	+0.00002	-0.00004	-0.00006
**4**	0.363	+0.00012	+0.00057	-0.00025	-0.00087
**5**	0.157	+0.00004	+0.00026	-0.00010	-0.00042
**6**	0.456	+0.00006	+0.00074	-0.00023	-0.00107
**7**	0.396	+0.00012	+0.00068	-0.00022	-0.00101
**8**	0.448	+0.00012	+0.00069	-0.00030	-0.00102
PHTHAC05	0.222	+0.00029	+0.00029	-0.00035	-0.00036
PODHUM	0.082	+0.00020	+0.00022	-0.00023	-0.00025
GECYEU	0.365	+0.00091	+0.00051	-0.00093	-0.00091
LEBRER	1.270	+0.00260	+0.00153	-0.00278	-0.00155
KOXBIK	0.390	+0.00107	+0.00031	-0.00158	-0.00027
CAJVIU	0.391	+0.00163	+0.00026	-0.00179	-0.00035
AZULUD	0.534	+0.00095	+0.00050	-0.00103	-0.00061
ZUKVUY	0.877	+0.00271	+0.00037	-0.00308	-0.00069
GAPDIK	0.260	+0.00004	+0.00059	-0.00012	-0.00064
LAGTIX	0.219	+0.00037	+0.00003	-0.00043	-0.00003
SUDAXAS01	0.094	+0.00074	+0.00010	-0.00073	-0.00024
JUHQEK	1.563	+0.00162	+0.00231	-0.00204	-0.00316
ACBZO01	0.401	+0.00016	+0.00028	-0.00024	-0.00040
WOCHIF	1.127	+0.00140	+0.00194	-0.00200	-0.00274
MODYIO	0.290	+0.00042	+0.00054	-0.00060	-0.00070
YEXQOH	0.198	+0.00000	+0.00054	-0.00029	-0.00077
CIQNEW	0.410	+0.00054	+0.00056	-0.00072	-0.00062
LUCHEY	0.627	+0.00145	+0.00055	-0.00208	-0.00082
BECLAW	1.411	+0.00273	+0.00108	-0.00357	-0.00139
DESPAT	1.087	+0.00061	+0.00236	-0.00077	-0.00287
GIRQAA*	0.777	+0.00117	+0.00078	-0.00165	-0.00104
PUFBEZ	0.222	+0.00004	+0.00038	-0.00017	-0.00070
JOSGIH*	0.029	+0.00001	-0.00001	-0.00010	-0.00005
IKAXII*	0.591	+0.00079	+0.00058	-0.00122	-0.00091
OPAKIA*	0.667	+0.00070	+0.00078	-0.00104	-0.00128
OMINII	0.429	+0.00075	+0.00043	-0.00111	-0.00069
XACLUK*	0.544	+0.00023	+0.00022	-0.00053	-0.00064
EZELOK01*	0.254	+0.00020	+0.00012	-0.00041	-0.00023

WIHKAB*	0.559	+0.00114	+0.00023	-0.00176	-0.00040
LOVNIT*	0.240	+0.00025	+0.00013	-0.00057	-0.00031
Ile-pro (135-136) PDB: 2opc	1.989	+0.00435	+0.00096	-0.00545	-0.00123
Lys-pro (50-51) PDB: 1k3i	0.767	+0.00126	+0.00019	-0.00169	-0.00052
Cys-pro (251-251) PDB: 1gcy	1.567	+0.00309	+0.00017	-0.00407	-0.00070
Leu-pro (379-380) PDB: 1g5a	0.748	+0.00170	+0.00022	-0.00227	-0.00039
Ile-pro (107-108) PDB: 1o7i	0.560	+0.00127	+0.00000	-0.00179	-0.00053
Val-pro (294-295) PDB: 1jnd	0.257	+0.00056	-0.00001	-0.00082	0.00010
Ala-pro (264-265) PDB: 2xu9	3.064	+0.00557	+0.00121	-0.00880	-0.00154
Thr-pro (3-4) PDB: 1fj2	0.539	+0.00073	+0.00052	-0.00121	-0.00102
Ile-pro (208-209) PDB: 1e2w	0.300	+0.00079	-0.00001	-0.00112	-0.00018
Leu-pro (20-21) PDB: 1a2p	0.832	+0.00170	+0.00058	-0.00253	-0.00082
Pro-pro (186-187) PDB:3cx2	1.113	+0.00255	-0.00001	-0.00356	-0.00027
Glu-pro (623-624) PDB:1eu1	0.270	+0.00063	0.000000	-0.00095	0.00000
Leu-Pro (141-142) PDB:2x5o	2.281	+0.00330	+0.00248	-0.00558	-0.00356
Ala-Pro (103-104) PDB:1g12	0.318	+0.00101	+0.00008	-0.00147	-0.00013
Pro-Ser (32-33) PDB:4psc	0.659	+0.00110	+0.00003	-0.00176	-0.00053
Thr-Glu (108-109) PDB:4pdy	0.790	+0.00140	+0.00005	-0.00244	-0.00044
His-Ser (331-332) PDB:1b6a	0.231	+0.00037	+0.00024	-0.00066	-0.00044
Ala-Asp (95-96) PDB:2bi8	0.551	+0.00105	+0.00039	-0.00184	+0.00003
Phe-Pro (76-77) PDB:1n08	0.466	+0.00123	+0.00011	-0.00221	-0.00005
Leu-Pro (110-111) PDB:1eb6	0.231	+0.00032	0.000000	-0.00064	-0.00017
Leu-Tyr (101-102) PDB:3u26	1.062	+0.00239	+0.00019	-0.00389	-0.00068
Ser-Asp (79-80) PDB:3ry4	0.624	+0.00120	-0.00040	-0.00207	+0.00008
Ala-phe (139-140) PDB:4y1w	0.651	+0.00117	+0.00008	-0.00211	-0.00049
Gln-Lys (9-10) PDB:3wcq	0.936	+0.00159	-0.00065	-0.00316	+0.00015
Thr-Thr (349-350) PDB:3uxf	0.356	+0.00055	-0.00001	-0.00113	-0.00030
Ala-Arg (119-120) PDB:1ejd	0.106	+0.00031	0.00000	-0.00062	0.00000
Phe-Gly (28-29) PDB:1odv	0.116	+0.00033	+0.00006	-0.00069	-0.00011
Asp-Pro (2-3) PDB:2vzp	0.106	+0.00037	+0.00003	-0.00071	-0.00009
Ala-Leu (388-389) PDB:1ikp	0.004	-0.00002	+0.00000	+0.00004	0.00000
Ala-Ala (402-403) PDB:3s5m	0.723	-0.00102	+0.00013	-0.00201	-0.00011

*For these molecules, calculations using 6-311+G(2d,p) basis set gave error. Therefore, the calculation for these molecules were performed using 6-311G(d) basis set.

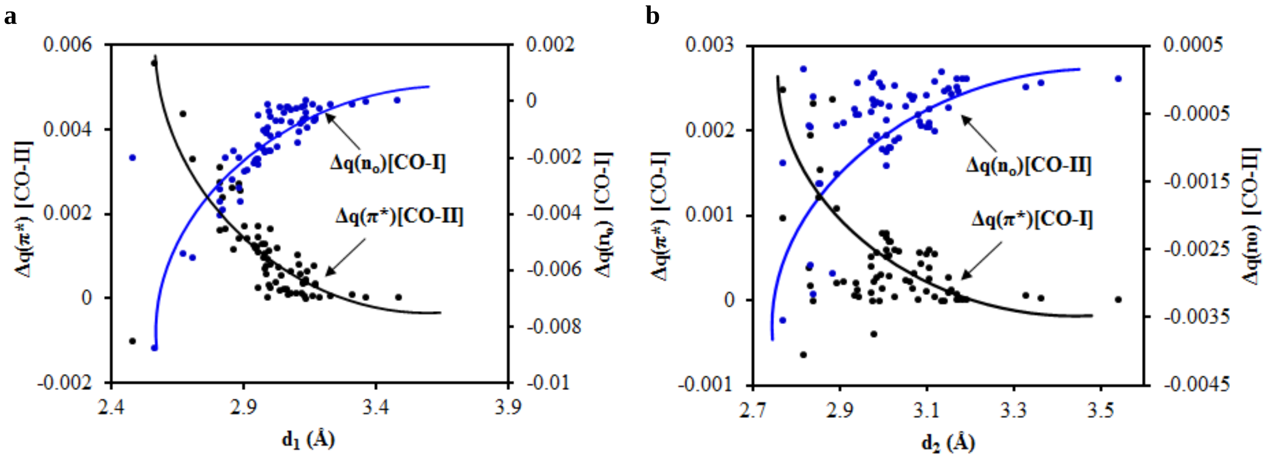

Supplementary Figure 10. Plots generated from NBO deletion data of $n \rightarrow \pi^*$ interactions. **a**, NBO deletion analysis carried out at HF/6-311+G(2d,p) level of theory show that as the strength of carbonyl-carbonyl short contact increases from CO-I to CO-II (d_1 decreases) there is an increase in the charge in $\pi^*_{C=O}$ orbital of CO-II [$\Delta q(\pi^*_{C=O})$] and decrease in the charge in the lone pair of oxygen in CO-I [$\Delta q(n_O)$] suggesting charge transfer via delocalization. The solid lines are drawn for convenience. **b**, NBO deletion analysis carried out at HF/6-311+G(2d,p) show that as the strength of carbonyl-carbonyl short contact increases from CO-II to CO-I (d_2 decreases) there is an increase in the charge in $\pi^*_{C=O}$ orbital of CO-I [$\Delta q(\pi^*_{C=O})$] and decrease in the charge in the lone pair of oxygen in CO-II [$\Delta q(n_O)$] suggesting charge transfer via delocalization. The solid lines are drawn for convenience. To generate plots (**a** and **b**), the d_1 and d_2 values are taken from Table 1-3 and $\Delta q(\pi^*_{C=O})$ and $\Delta q(n_O)$ values are taken from Supplementary Table 13.

Supplementary Table 14. NBO deletion analyses carried out on crystal geometries at HF/6-311+G(2d,p) level of theory. ΔE_{del} is the NBO deletion energy for the orbital interaction energy between the donor carbonyl $\pi_{C=O}$ orbital and the $\pi^*_{C=O}$ orbital of the acceptor carbonyl. $\Delta q(\pi^*)$ is the increase in the charge of the $\pi^*_{C=O}$ orbital due to the orbital interaction with the donor carbonyl $\pi_{C=O}$ orbital. $\Delta q(\pi)$ is the decrease in charge in the donor carbonyl $\pi_{C=O}$ orbital due to the orbital interaction with the acceptor $\pi^*_{C=O}$ orbital.

**Compound**	**ΔE_{del}**	**$\Delta q(\pi^*)$**		**$\Delta q(\pi)$**	
	**(kcal.mol^{-1})**	**(electron)**		**(electron)**	
		**COI to COII**	**COII to COI**	**COI to COII**	**COII to COI**
**1**	0.032	+0.00015	+0.00012	-0.00014	-0.00011
**2**	0.034	+0.00012	+0.00013	-0.00013	-0.00013
**3**	0.036	+0.00006	+0.00008	-0.00008	-0.00008
**4**	0.041	-0.00007	0.00000	+0.00005	0.00000
**5**	-0.004	+0.00001	+0.00002	0.00000	-0.00003
**6**	0.030	-0.00004	+0.00001	+0.00003	0.00000
**7**	0.050	-0.00007	-0.00002	+0.00007	+0.00001
**8**	0.042	-0.00008	0.00000	+0.00007	+0.00003
PHTHAC05	-	-	-	-	-
PODHUM	-	-	-	-	-
GECYEU	-	-	-	-	-
LEBRER	0.012	0.00000	+0.00001	-0.00002	+0.00001
KOXBIK	-	-	-	-	-
CAJVIU	0.000	-0.00005	+0.00007	0.00000	-0.00009
AZULUD	0.008	+0.00005	+0.00007	-0.00002	-0.00005
ZUKVUY	0.336	+0.00002	+0.00006	-0.00009	-0.00032
GAPDIK	0.020	+0.00015	0.0000	-0.00014	+0.00001
LAGTIX	-	-	-	-	-
SUDAXAS01	-0.132	0.00000	+0.00004	+0.00001	-0.00008
JUHQEK	0.296	+0.00027	+0.00045	-0.00022	-0.00038
ACBZO01	0.039	+0.00008	+0.00009	-0.00005	-0.00006
WOCHIF	0.466	+0.00067	+0.00081	-0.00074	-0.00089
MODYIO	0.089	+0.00019	+0.00023	-0.00021	-0.00024
YEXQOH	0.009	0.00000	0.00000	-0.00002	-0.00000
CIQNEW	0.051	+0.00018	+0.00007	-0.00015	-0.00004
LUCHEY	0.119	+0.00012	+0.00016	-0.00014	-0.00014
BECLAW	0.466	+0.00053	+0.00018	-0.00062	-0.00006
DESPAT	0.608	+0.00053	+0.00115	-0.00058	-0.00121
GIRQAA*	0.446	+0.00061	+0.00033	-0.00063	-0.00031
PUFBEZ	0.083	+0.00002	+0.00014	-0.00008	-0.00019
JOSGIH*	0.157	+0.00008	-0.00002	-0.00041	-0.00002
IKAXII*	0.885	+0.00117	+0.00107	-0.00138	-0.00120
OPAKIA*	0.926	+0.00119	+0.00128	-0.00133	-0.00151
OMINII	0.442	+0.00095	+0.00067	-0.00104	-0.00075
XACLUK*	2.158	+0.00289	+0.00304	-0.00350	-0.00377

EZELOK01*	1.323	+0.00163	+0.00117	-0.00193	-0.00140
WIHKAB*	1.012	+0.00120	+0.00148	-0.00176	-0.00170
LOVNIT*	1.482	+0.00172	+0.00130	-0.00251	-0.00194
Ile-pro (135-136) PDB: 2opc	0.223	-0.00022	+0.00004	-0.0004	-0.00007
Lys-pro (50-51) PDB: 1k3i	-	-	-	-	-
Cys-pro (251-251) PDB: 1gcy	0.151	+0.00008	+0.00013	-0.00006	-0.00016
Leu-pro (379-380) PDB: 1g5a	0.091	+0.00001	+0.00007	-0.00002	-0.00007
Ile-pro (107-108) PDB: 1o7i	0.040	+0.00015	0.00000	-0.00015	-0.00006
Val-pro (294-295) PDB: 1jnd	0.030	+0.00001	+0.00006	0.00000	-0.00008
Ala-pro (264-265) PDB: 2xu9	0.239	+0.00021	+0.00014	-0.00035	-0.00004
Thr-pro (3-4) PDB: 1fj2	0.040	+0.00017	+0.00002	-0.00018	0.00000
Ile-pro (208-209) PDB: 1e2w	0.038	+0.00001	+0.00004	0.00000	-0.00009
Leu-pro (20-21) PDB: 1a2p	0.189	+0.00020	+0.00017	-0.00024	-0.00016
Pro-pro (186-187) PDB:3cx2	0.200	+0.00010	+0.00012	-0.00023	-0.00013
Glu-pro (623-624) PDB:1eu1	0.030	-0.00002	+0.00002	-0.00003	-0.00003
Leu-Pro (141-142) PDB:2x5o	1.415	+0.00159	+0.00072	-0.00166	-0.00103
Ala-Pro (103-104) PDB:1g12	0.118	+0.00011	+0.00007	-0.00014	-0.00012
Pro-Ser (32-33) PDB:4psc	0.355	+0.00051	+0.00035	-0.00062	-0.00053
Thr-Glu (108-109) PDB:4pdy	0.472	+0.00046	+0.00030	-0.00068	-0.00053
His-Ser (331-332) PDB:1b6a	0.248	+0.00029	+0.00023	-0.00041	-0.00037
Ala-Asp (95-96) PDB:2bi8	0.378	+0.00041	+0.00010	-0.00056	-0.00040
Phe-Pro (76-77) PDB:1n08	0.418	+0.00042	+0.00028	-0.00057	-0.00055
Leu-Pro (110-111) PDB:1eb6	0.056	-0.00002	+0.00002	-0.00001	-0.00015
Leu-Tyr (101-102) PDB:3u26	1.000	+0.00125	+0.00062	-0.00169	-0.00083
Ser-Asp (79-80) PDB:3ry4	0.409	+0.00049	-0.00002	-0.00068	-0.00030
Ala-phe (139-140) PDB:4y1w	0.725	+0.00082	+0.00041	-0.00117	-0.00071
Gln-Lys (9-10) PDB:3wcq	1.376	+0.00083	-0.00025	-0.00150	-0.00089
Thr-Thr (349-350) PDB:3uxf	0.614	+0.00066	+0.00037	-0.00103	-0.00076
Ala-Arg (119-120) PDB:1ejd	0.221	+0.00023	-0.00014	-0.00039	-0.00010
Phe-Gly (28-29) PDB:1odv	0.409	+0.00036	+0.00001	-0.00078	-0.00032
Asp-Pro (2-3) PDB:2vzp	0.712	+0.00019	+0.00110	-0.00043	-0.00222
Ala-Leu (388-389) PDB:1ikp	0.004	0.00000	-0.00001	+0.00001	0.00000
Ala-Ala (402-403) PDB:3s5m	3.190	+0.00080	+0.00036	-0.00418	-0.00069

*For these molecules, calculations using 6-311+G(2d,p) basis set gave error. Therefore, the calculation for these molecules were performed using 6-311G(d) basis set.

a

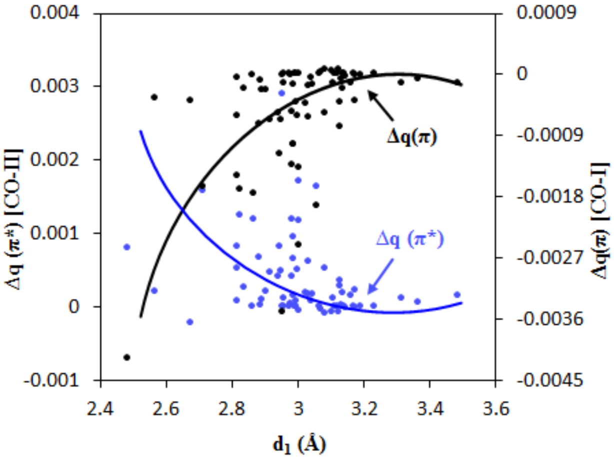

b

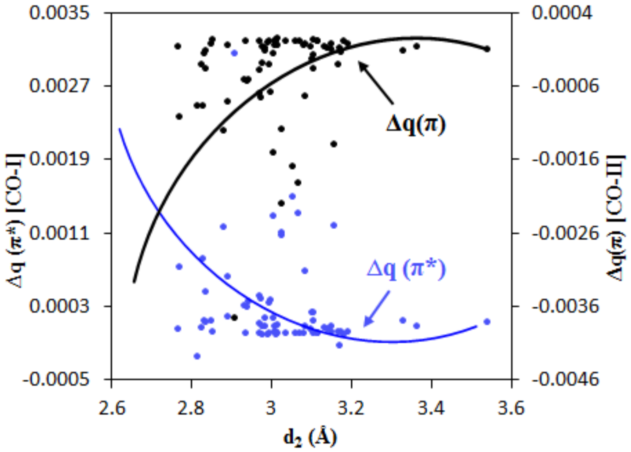

Supplementary Figure 11. Plots generated from NBO deletion data of $\pi \rightarrow \pi^*$ interactions. **a**, NBO deletion analysis carried out at HF/6-311+G(2d,p) level of theory show that as the strength of carbonyl-carbonyl short contact increases from CO-I to CO-II (d_1 decreases) there is an increase in the charge in the $\pi^*_{C=O}$ orbital of CO-II [$\Delta q(\pi^*)$] and decrease in the charge in $\pi_{C=O}$ orbital of CO-I [$\Delta q(\pi)$] suggesting charge transfer via delocalization. The solid lines are drawn for convenience. **b**, NBO deletion analysis carried out at HF/6-311+G(2d,p) show that as the strength of carbonyl-carbonyl short contact increases from CO-II to CO-I (d_2 decreases) there is an increase in the charge in $\pi^*_{C=O}$ orbital of CO-I [$\Delta q(\pi^*_{C=O})$]and decrease in charge in $\pi_{C=O}$ orbital of CO-II [$\Delta q(\pi)$] suggesting charge transfer via delocalization. The solid lines are drawn for convenience. To generate plots (**a** and **b**), the d_1 and d_2 values are taken from Table 1-3 and $\Delta q(\pi^*)$ and $\Delta q(\pi)$ values are taken from Supplementary Table 14.

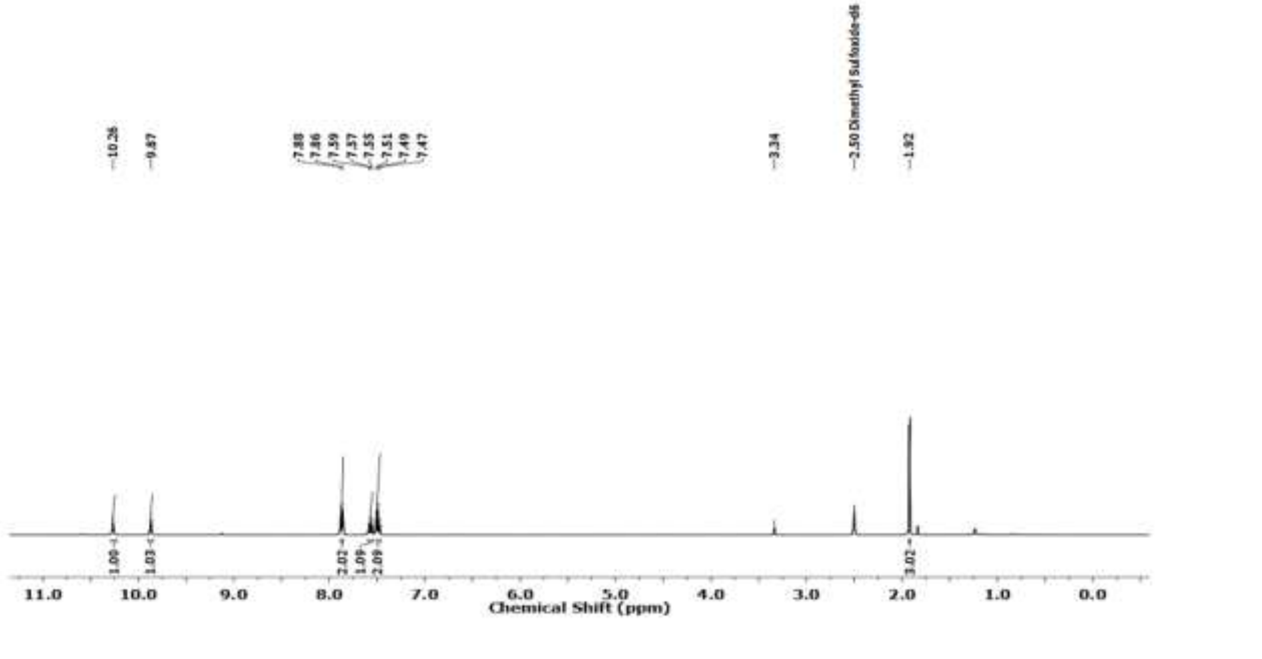

Supplementary Figure 12. ^{1}H NMR of compound **1**.

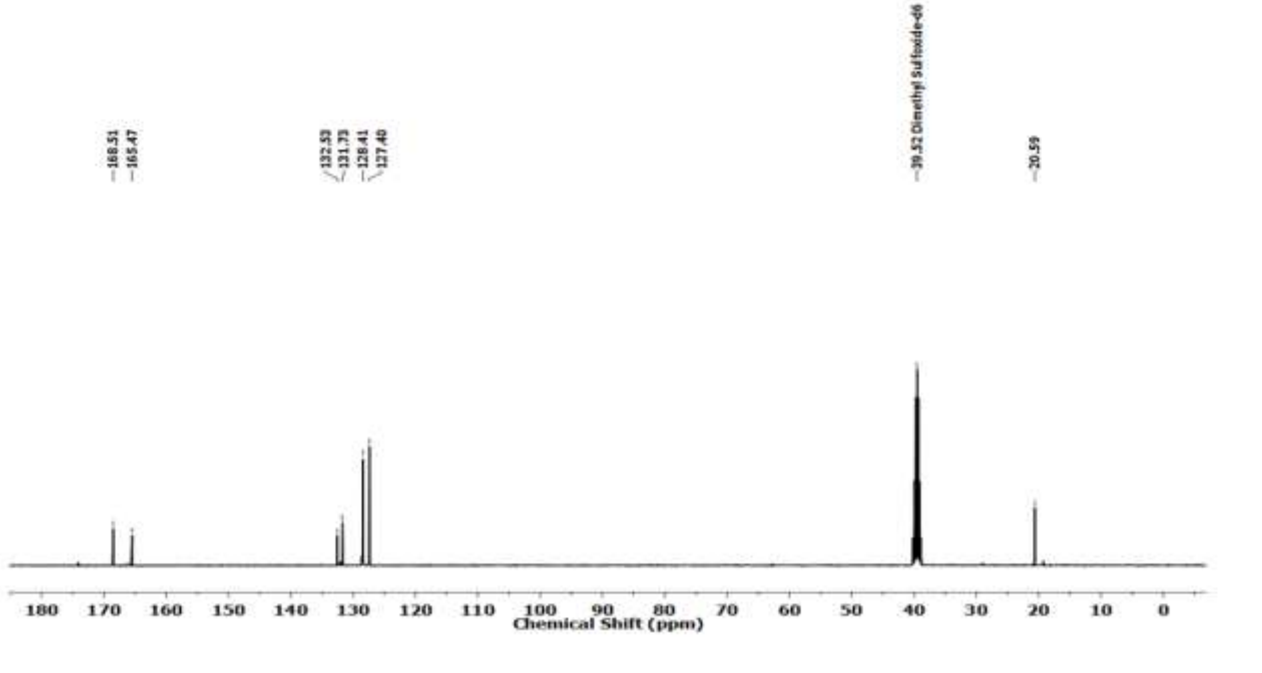

Supplementary Figure 13. ^{13}C NMR of Compound **1**.

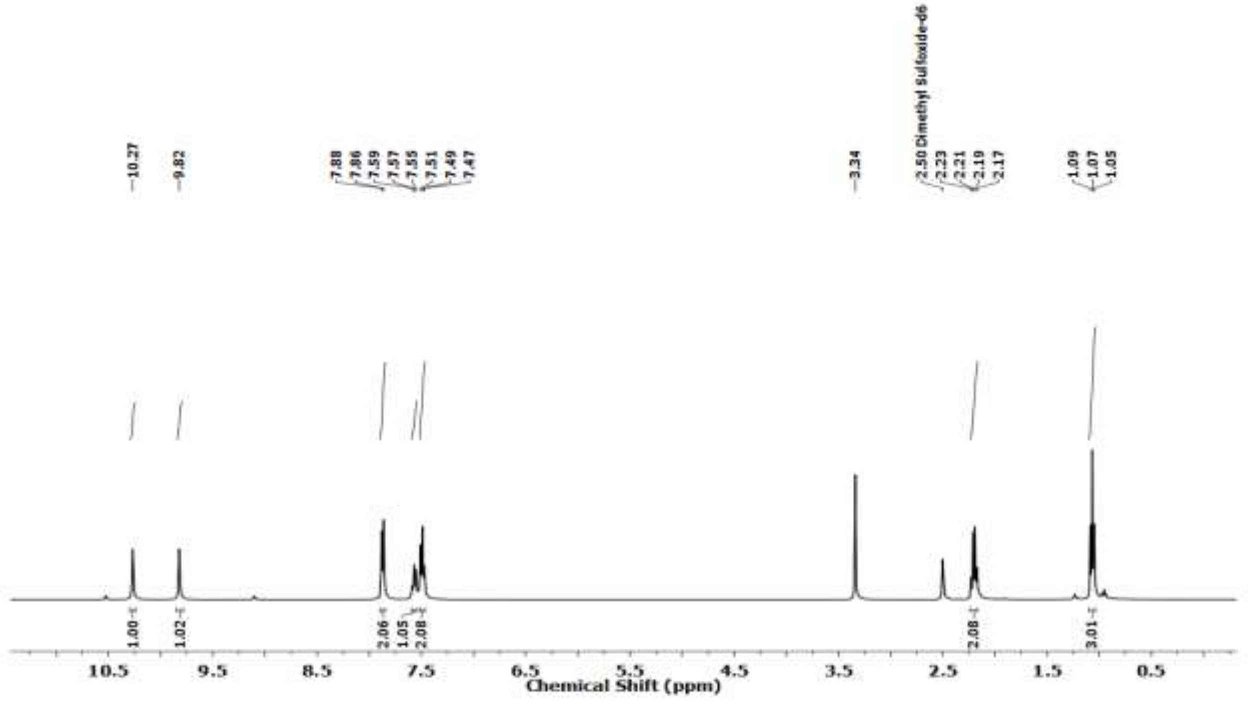

Supplementary Figure 14. ^{1}H NMR of Compound 2.

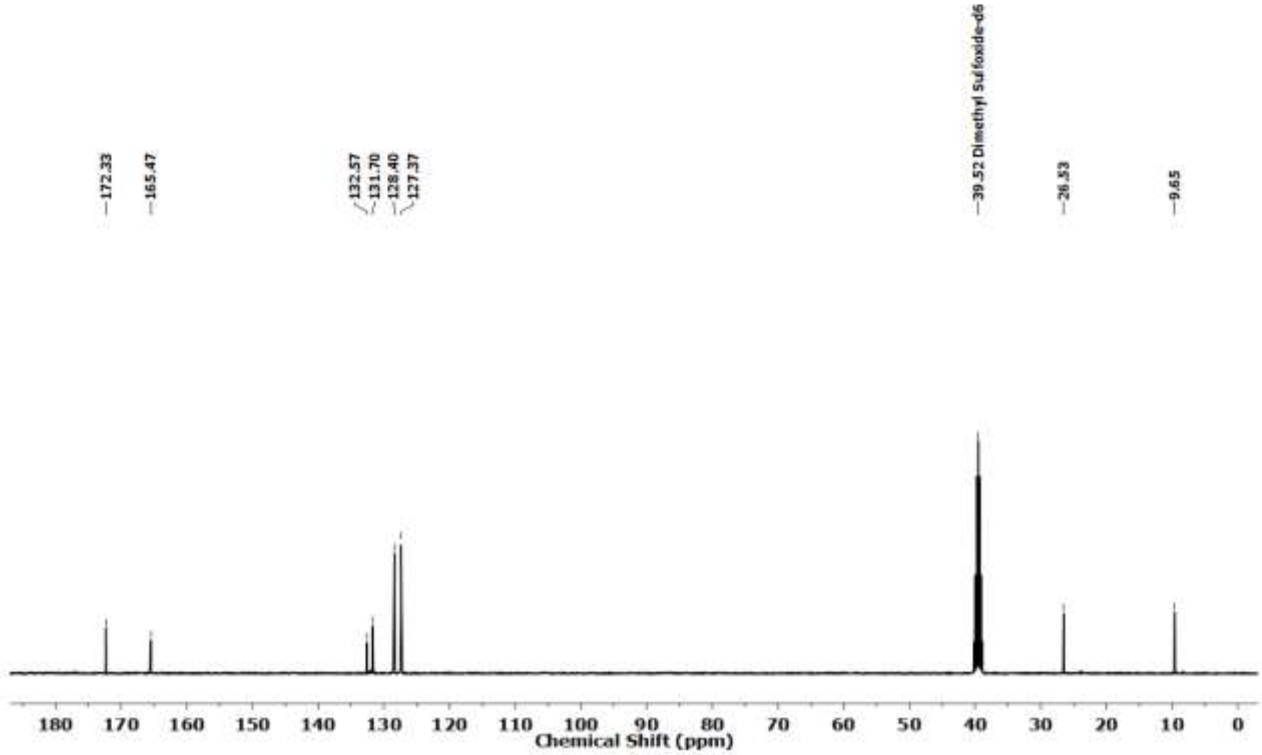

Supplementary Figure 15. ^{13}C NMR of Compound **2**.

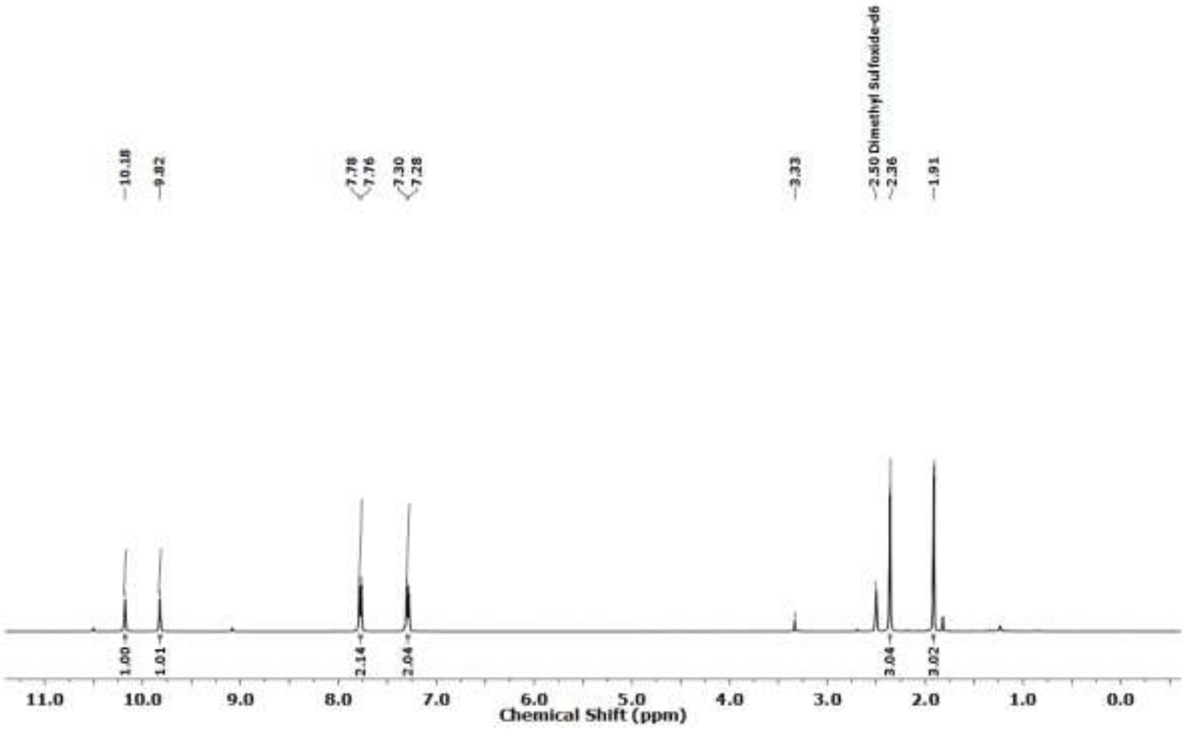

Supplementary Figure 16. ^{1}H NMR of Compound **3**.

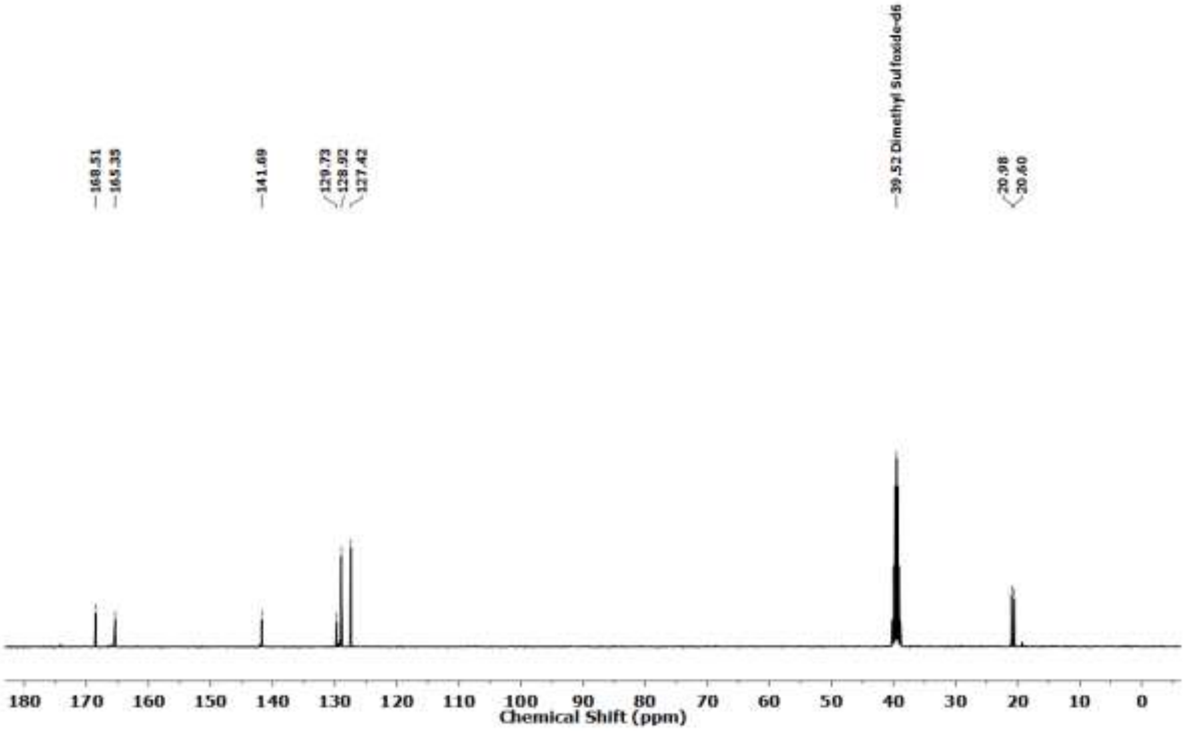

Supplementary Figure 17. ^{13}C NMR of Compound **3.**

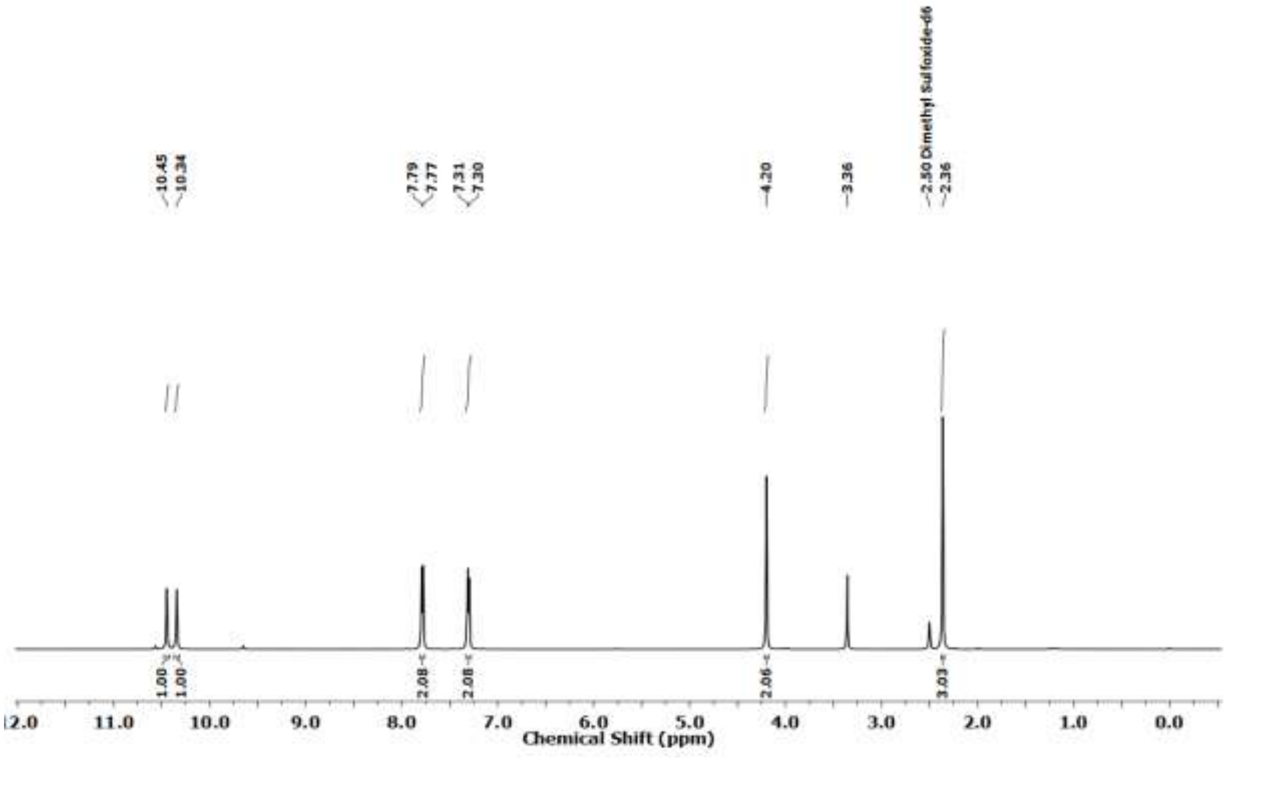

Supplementary Figure 18. ^{1}H NMR of Compound **4**.

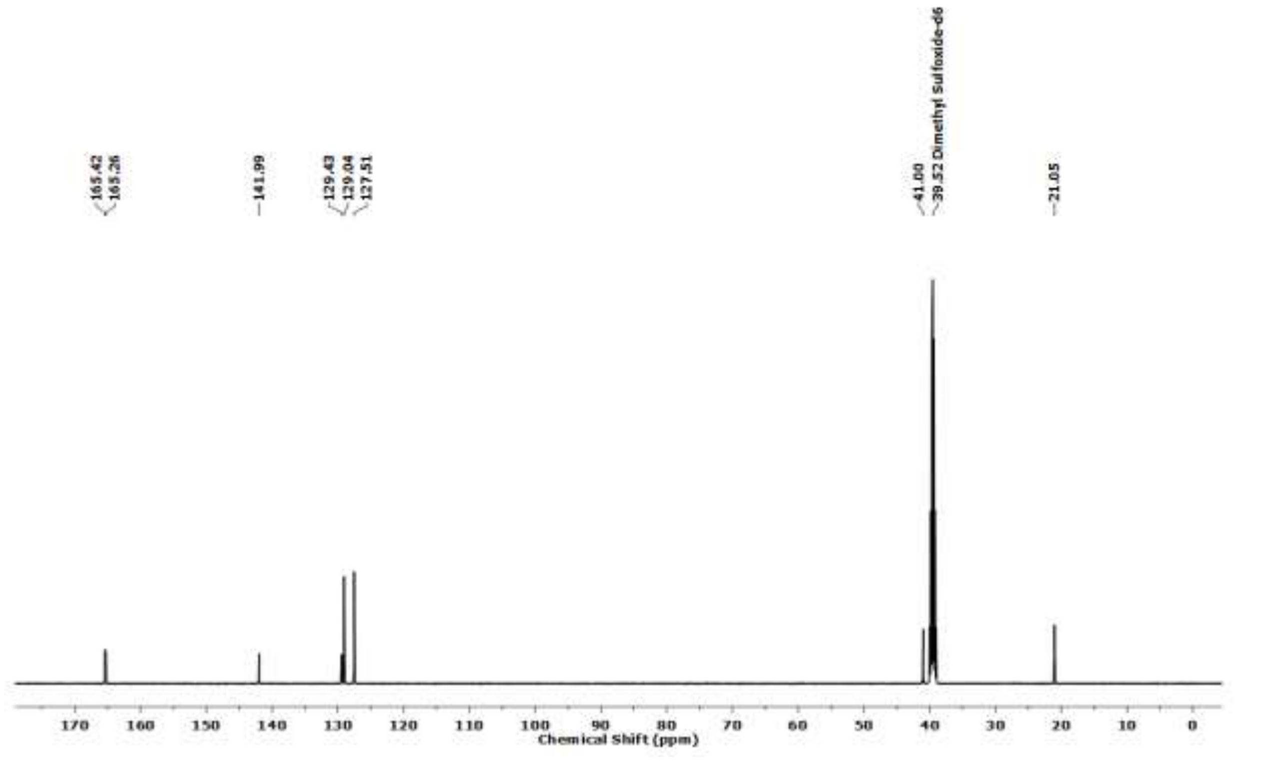

Supplementary Figure 19. ^{13}CNMR of Compound **4**.

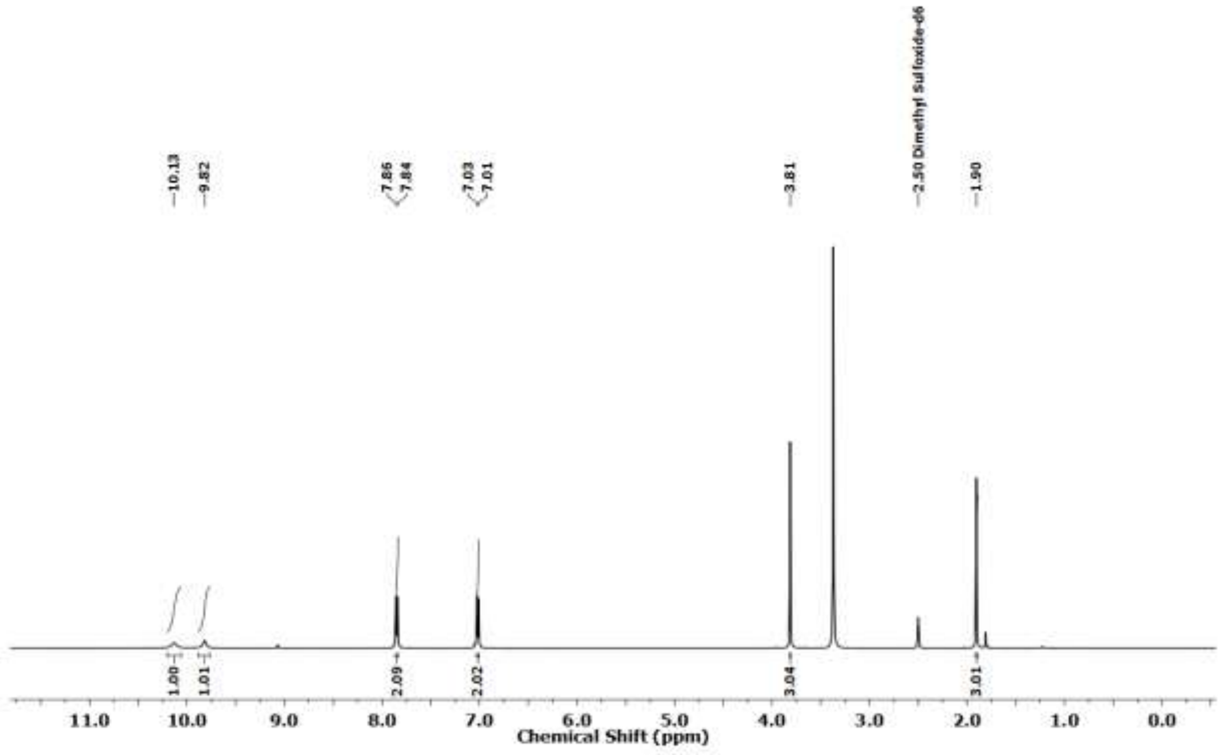

Supplementary Figure 20. ^{1}H NMR of Compound **5**.

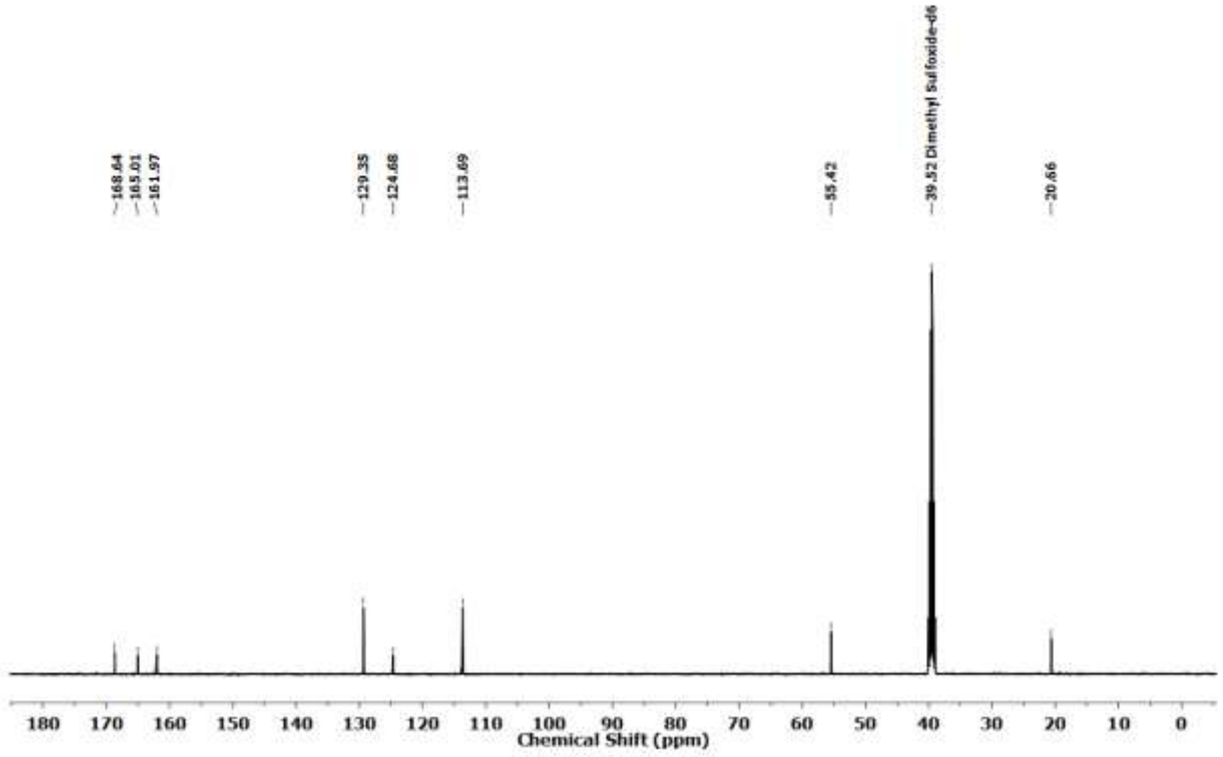

Supplementary Figure 21. ^{13}C NMR of Compound **5**.

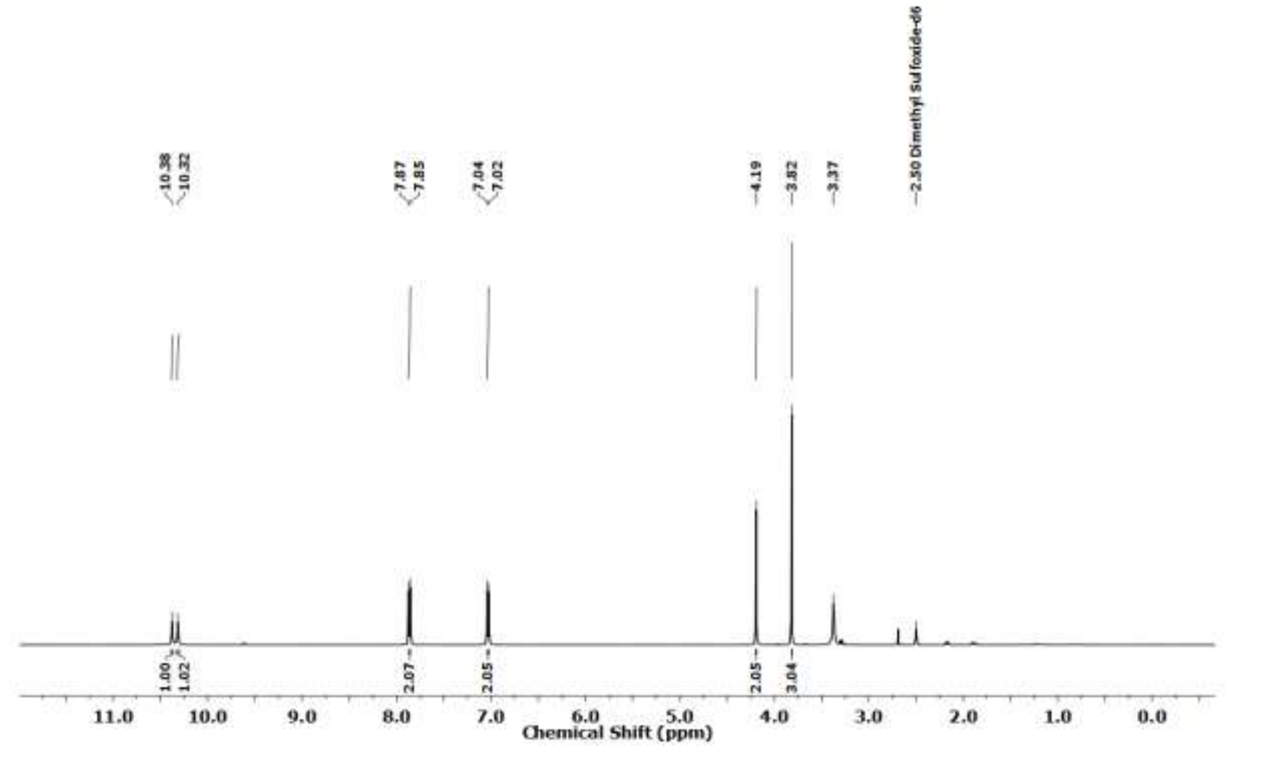

Supplementary Figure 22. ^{1}H NMR of Compound **6**.

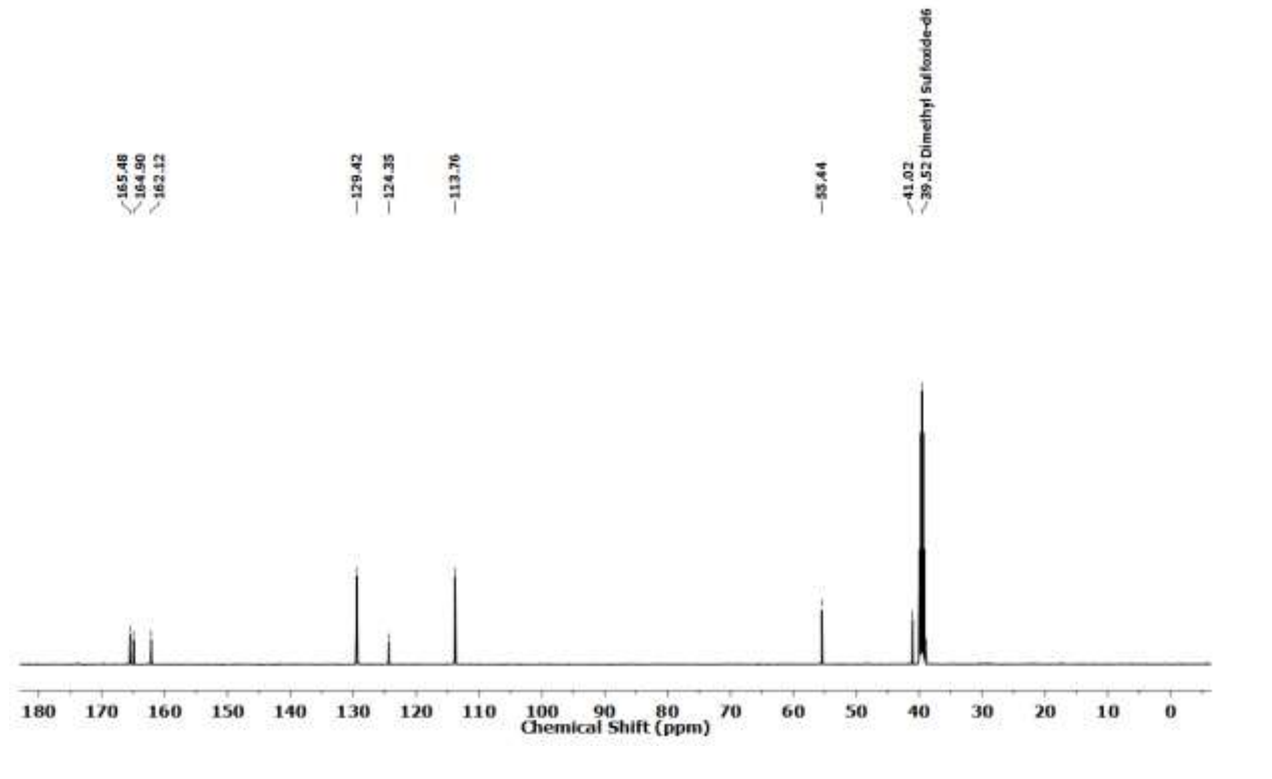

Supplementary Figure 23. ^{13}C NMR of Compound **6**.

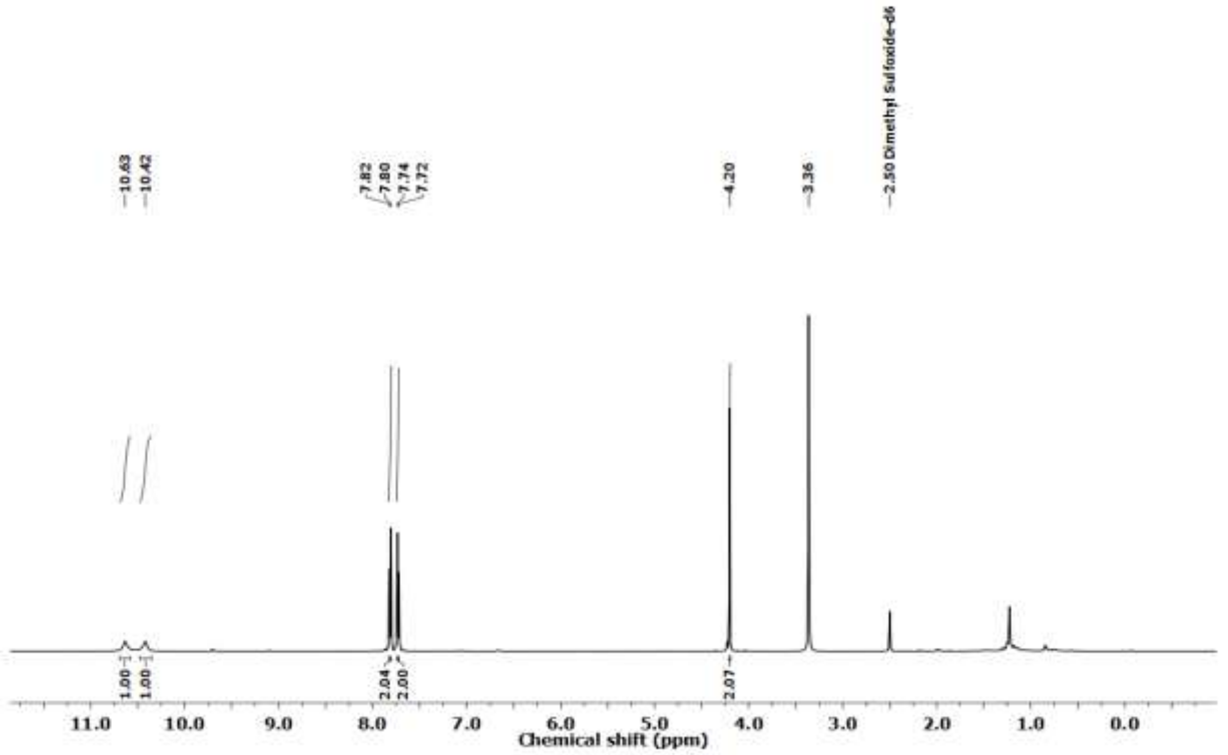

Supplementary Figure 24. ^{1}H NMR of Compound **7**.

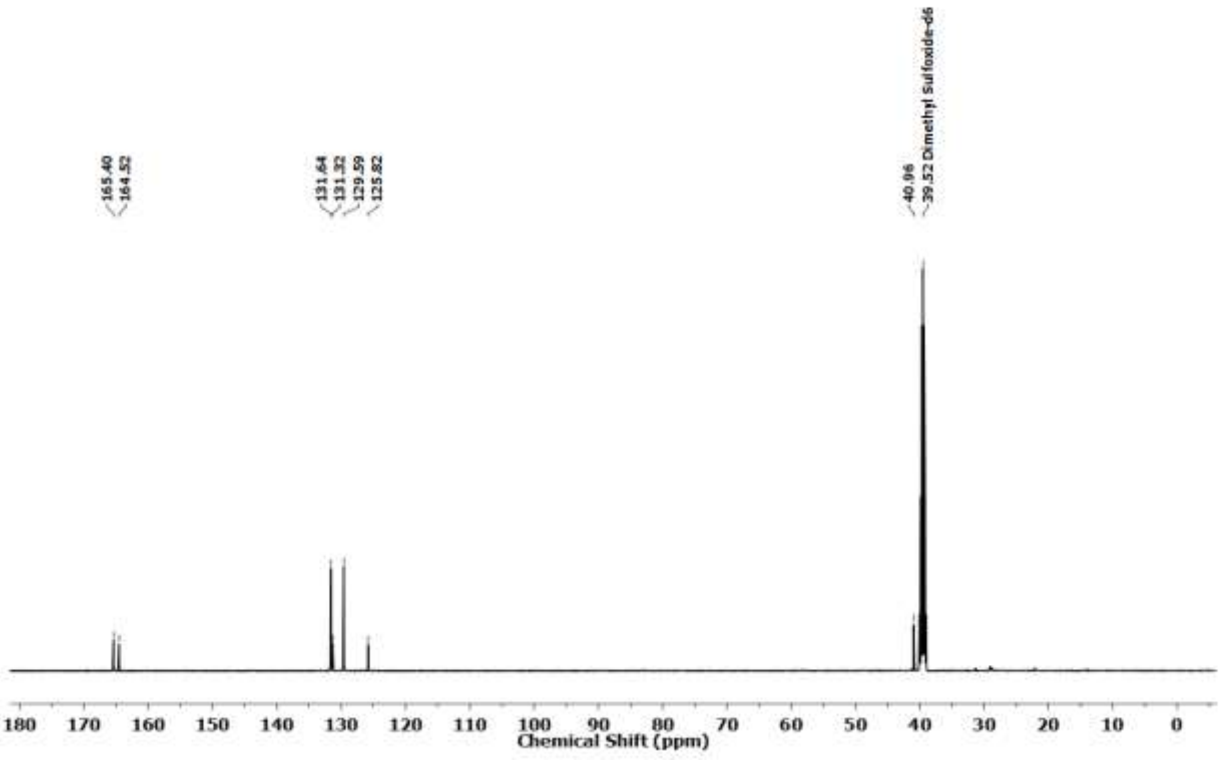

Supplementary Figure 25. ^{13}C NMR of Compound **7**.

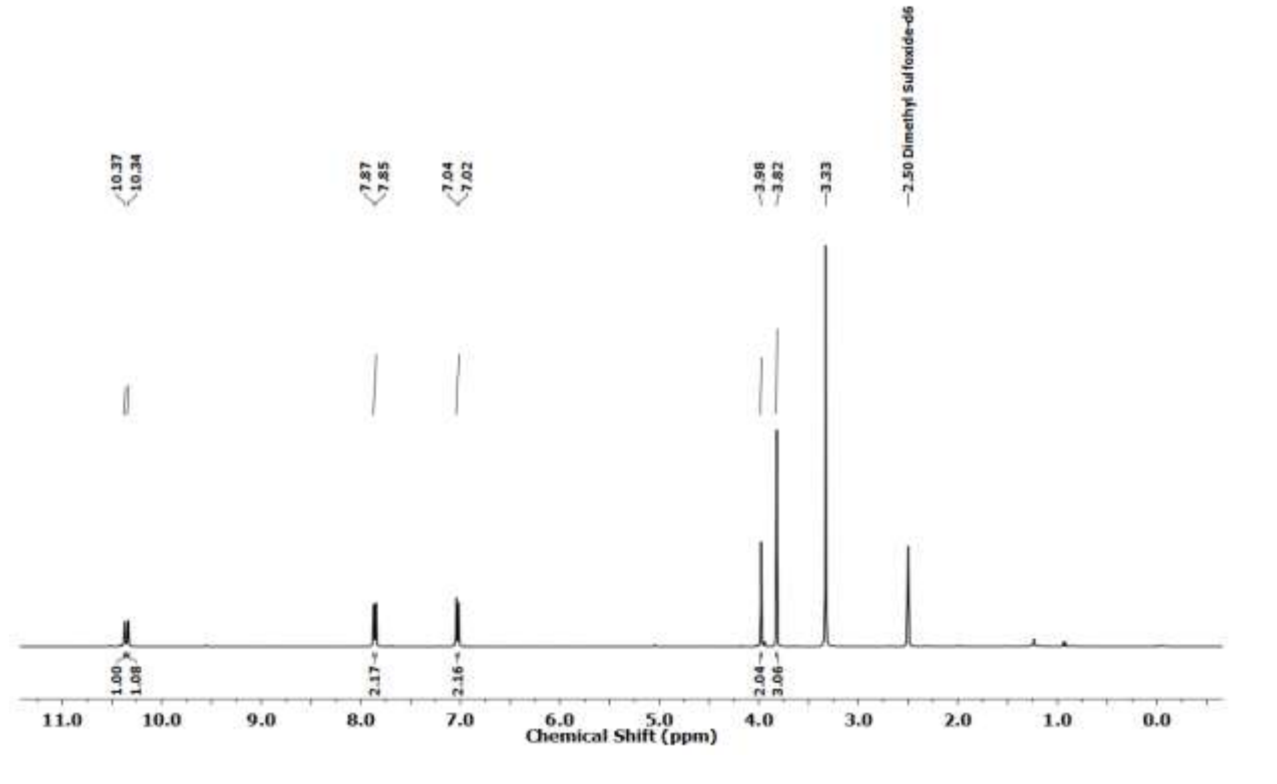

Supplementary Figure 26. ^{1}H NMR of Compound **8**.

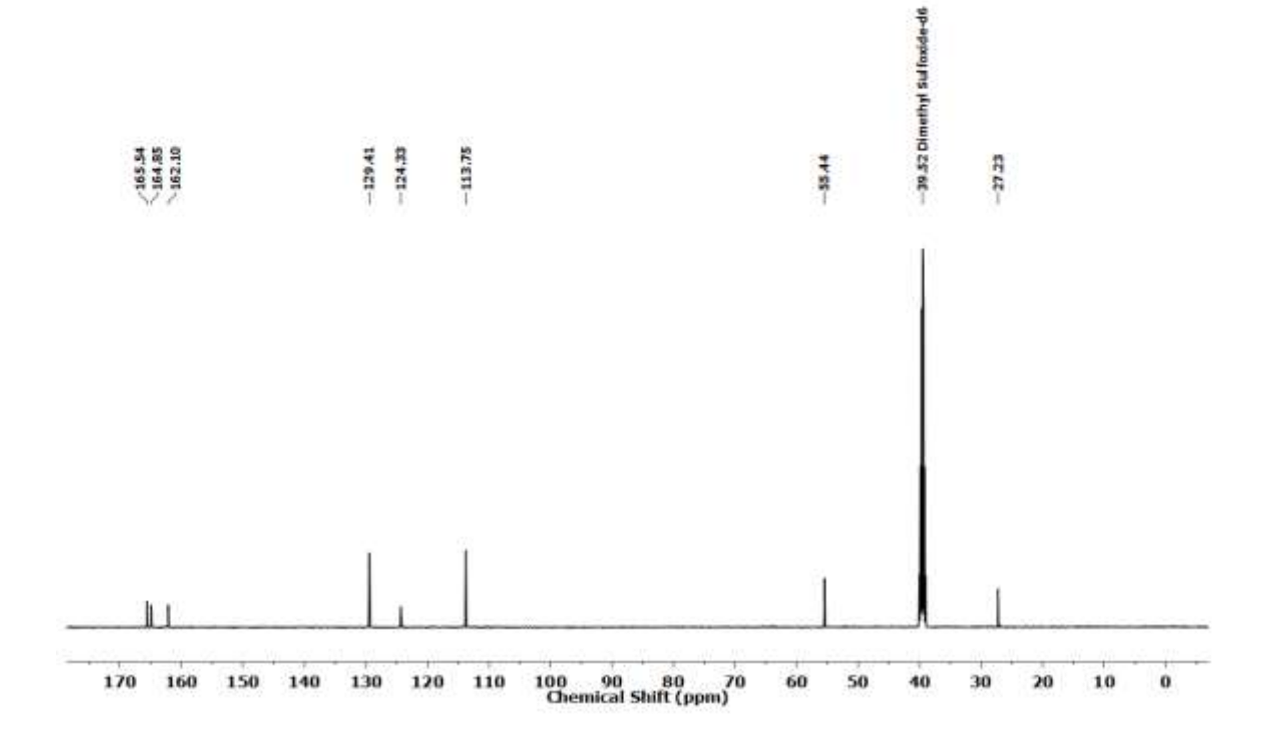

Supplementary Figure 27. ^{13}C NMR of Compound **8**.
